# Genome-Wide Identification of HrpL-Regulated Genes in the Necrotrophic Phytopathogen *Dickeya dadantii* 3937

**DOI:** 10.1371/journal.pone.0013472

**Published:** 2010-10-19

**Authors:** Shihui Yang, Quan Peng, Qiu Zhang, Lifang Zou, Yan Li, Christelle Robert, Leighton Pritchard, Hui Liu, Raymond Hovey, Qi Wang, Paul Birch, Ian K. Toth, Ching-Hong Yang

**Affiliations:** 1 Department of Biological Sciences, University of Wisconsin, Milwaukee, Wisconsin, United States of America; 2 Department of Plant Pathology, China Agricultural University, Beijing, China; 3 Plant Pathology, Scottish Crop Research Institute, Invergowrie, Dundee, United Kingdom; University College Dublin, Ireland

## Abstract

**Background:**

*Dickeya dadantii* is a necrotrophic pathogen causing disease in many plants. Previous studies have demonstrated that the type III secretion system (T3SS) of *D. dadantii* is required for full virulence. HrpL is an alternative sigma factor that binds to the *hrp* box promoter sequence of T3SS genes to up-regulate their expression.

**Methodology/Principal Findings:**

To explore the inventory of HrpL-regulated genes of *D. dadantii* 3937 (3937), transcriptome profiles of wild-type 3937 and a *hrpL* mutant grown in a T3SS-inducing medium were examined. Using a cut-off value of 1.5, significant differential expression was observed in sixty-three genes, which are involved in various cellular functions such as type III secretion, chemotaxis, metabolism, regulation, and stress response. A hidden Markov model (HMM) was used to predict candidate *hrp* box binding sites in the intergenic regions of 3937, including the promoter regions of HrpL-regulated genes identified in the microarray assay. In contrast to biotrophic phytopathgens such as *Pseudomonas syringae,* among the HrpL up-regulated genes in 3937 only those within the T3SS were found to contain a *hrp* box sequence. Moreover, direct binding of purified HrpL protein to the *hrp* box was demonstrated for *hrp* box-containing DNA fragments of *hrpA* and *hrpN* using the electrophoretic mobility shift assay (EMSA). In this study, a putative T3SS effector DspA/E was also identified as a HrpL-upregulated gene, and shown to be translocated into plant cells in a T3SS-dependent manner.

**Conclusion/Significances:**

We provide the genome-wide study of HrpL-regulated genes in a necrotrophic phytopathogen (*D. dadantii* 3937) through a combination of transcriptomics and bioinformatics, which led to identification of several effectors. Our study indicates the extent of differences for T3SS effector protein inventory requirements between necrotrophic and biotrophic pathogens, and may allow the development of different strategies for disease control for these different groups of pathogens.

## Introduction

The type III secretion system (T3SS) is an essential virulence determinant of many bacterial pathogens that delivers effector proteins into host tissues. In plant pathogens such as *Pseudomonas* spp., *Dickeya* spp., *Erwinia* spp., *Pantoea* spp., *Ralstonia* spp., and *Xanthomonas* spp., the T3SS is required for bacterial pathogenicity on host plants and for elicitation of the hypersensitive response (HR), a localized programmed cell death, in non-host plants [1]–[4]. The T3SS genes of phytobacteria are induced in the plant apoplast, when in close contact with host cells or in nutritionally poor synthetic T3SS-inducing minimal medium, which is thought to mimic conditions within the plant apoplast [1], [2], [4].

Expression of the T3SS in bacteria is coordinately regulated by networks of transcription factors in response to environmental stimuli. Based on differences in regulation, T3SSs can be divided into two groups. The Group I system is found in *P. syringae*, *Dickeya, Erwinia*, and *Pantoea* spp., where T3SS genes are activated by HrpL, a member of the extracytoplasmic factor (ECF) family alternative sigma factor [5], [6]. The Group II system is found in *X. campestris* and *R. solanacearum*, where transcription of T3SS-associated genes is regulated by members of the AraC family of transcriptional proteins [4], [6]–[8].

The T3SS regulatory pathway of *D. dadantii* 3937 (3937) has recently been elucidated [9]–[13]. Similar to *E. amylovora* and *P. syringae*, the T3SS of 3937 is regulated by a two-component signal system (TCS) HrpX/HrpY, which activates the gene encoding HrpS, a σ^54^-enhancer binding protein. The HrpS protein is required for expression of the alternative sigma factor, *hrpL*. Presumably, HrpL of 3937 up-regulates several T3SS genes downstream in the type III secretion regulon. In 3937, the T3SS genes are also regulated by an alternative pathway in which the RsmA protein down-regulates T3SS genes by affecting the mRNA stability and translation of *hrpL*. Another TCS GacS/GacA activates an untranslated regulatory RNA *rsmB*, which sequesters RsmA and neutralizes its effect on *hrpL* [10], [11], [14].

Different modes of infection are used by phytopathogens to invade plant hosts. Biotrophic phytopathogens, such as *P. syringae*, keep the host plant cells alive and rely on living cells for growth and tissue colonization. Necrotrophic pathogens such as *D. dadantii*, on the other hand, kill plant cells upon tissue colonization by producing toxic proteins or plant cell wall degrading enzymes (PCWDEs), including pectinases [15]–[18]. Although it is becoming clear that phytopathogens may engage in either or both of these processes at different times of the infection process, for convenience the above, rather strict, definitions will be used throughout the manuscript [19].

In most biotrophic phytopathogens, T3SS deficient mutants are significantly attenuated in virulence [2], [5], [6], [20]–[22]. However, due to the production of PCWDEs, T3SS mutants of 3937 still cause maceration but are delayed in initial multiplication within the plant [17], [23].

To further expand our knowledge in this area, microarray and bioinformatics analyses were used to investigate HrpL-controlled genes within 3937 on a genome-wide scale, and the interaction of the HrpL protein with promoter regions of T3SS genes was further studied. In addition, the ability of the T3SS to translocate the 3937 effector protein DspA/E was demonstrated using a calmodulin-dependent adenylate cyclase (Cya) as a reporter.

## Results

### Use of transcriptomics to identify HrpL-regulated genes

Although HrpL is considered to be the major sigma factor controlling many T3SS genes in several biotrophic phytopathogens, limited information is available describing the mechanism by which HrpL, the alternative sigma factor of a necrotrophic pathogen such as *D. dadantii* regulates T3SS gene expression. To explore the inventory of HrpL-regulated genes in 3937, the transcriptome profile differences between wild-type 3937 and a *hrpL* mutant WPP96 (Supplementary Table S1) were compared by microarray analysis. The microarray results are based on the geomean of ten normalized expression values derived from three biological replicates and two dye-swap experiments with a technical replicate for each array.

Based on our microarray assays, we identified 63 genes that were differentially regulated between 3937 and WPP96. Approximately 40% of these genes were up-regulated by HrpL with the arbitrary cut-off value of 1.5-fold difference (Table 1 and Table 2; Supplementary Fig. S1, S2). The HrpL-up-regulated T3SS genes are classified into two major categories; 1) T3SS substrate genes *dspA/E, hrpA, hrpK, hrpN,* and *hrpW*; and 2) T3SS apparatus genes *hrpP*, *hrc*Q, *hrp*G, and *hrpF*. In addition, *orfC* (a potential *hrpW* chaperone), and two proteins of unknown function located in the *hrp* cluster were also up-regulated by HrpL (Table 1). Other HrpL up-regulated genes identified in the microarray assay included those with functions related to regulation and chemotaxis, as well as others with unknown function (Table 1; Supplementary Fig. S1). In our microarray assay, two thirds of the HrpL-down-regulated genes have unknown functions, and the remainder are possibly involved in regulation, transportation, metabolism, or stress response (Table 2; Supplementary Fig. S1).

**Table 1:** HrpL up-regulated genes in *Dickeya dadantii* 3937.

ASAP ID	Gene	Product	Microarray Ratio a	qRT-PCR Ratio^c^	*hrp* box d
**T3SS**					
**15579**	***hrpJ***	**HrpJ**	**2.21** b		**+**
15584	*hrpP*	HrpP	1.98 b		
**15585**	***hrcQ***	**HrcQ**	**2.12** b		
**19004**	***hrpK***	**HrpK**	**2.07** b	**7.22**±**2.29** *	**+**
**19006**	***orfC***	**HrpW chaperone**	**2.53** b	**7.76**±**2.02** *	
**19007**		**Unknown protein**	**2.67** b		
**19008**		**Unknown protein**	**2.35** b		
19009	*hrpW*	HrpW	1.94 b		+
19012	*dspA/E*	DspA/E	1.66	200.12±82.82^e^ *	
**19593**	***hrpA***	**HrpA**	**13.33**		**+**
**20784**	***hrpN***	**HrpN**	**9.08**		**+**
20865	*hrpG*	HrpG	1.74 b		
**20866**	***hrpF***	**HrpF**	**2.17** b		**+**
**Regulator**					
18408	*dcuS*	Signal transduction histidine kinase	1.83		
**Metabolism**					
17589	*cyoE*	Protoheme IX farnesyltransferase (haeme O biosynthesis)	1.60	1.28±0.67	
**Transporter**					
**15704**		**Drug resistance transporter Bcr/CflA subfamily**	**2.00**	**0.56**±**0.13**	
**Chemotaxis**					
17896		Methyl-accepting chemotaxis protein	1.69		
18765		Methyl-accepting chemotaxis protein	1.51		
**Unknown**					
14661		Unknown protein	1.50		
15855		Conserved unknown protein	1.55		
17664		Unknown protein	1.70		
18251		Conserved unknown protein	1.52		
18705		Unknown protein	1.78		
19405		Unknown protein	1.51		

a : Ratio for 3937 wild type/*hrpL* mutant is the geomean of ten arrays (five slides, each slide has two arrays) from three biological replicates. HrpL up-regulated genes have the transcript abundances differing by 1.5-fold and SAM q-value less than 1% in minimal medium 6 h post inoculation at 28°C. Genes in bold font had at least a 2-fold change.

b : Several genes in one operon or overlapped genes are regulated.

c : The ratio of qRT-PCR from three replicates.

d : Hrp box was predicted with a hidden Markov model (HMM).

e : Ct value of qRT-PCR for *dspA/E* in *hrpL* mutant is 35 and 27 in wild-type strain 3937.

*: Significant difference determined by student t test (*p*<0.05).

**Table 2:** HrpL down-regulated genes in *Dickeya dadantii* 3937.

ASAP ID	Gene	Product	Microarray Ratio a	qRT-PCR Ratio^c^	*hrp*box^d^
**Stress**					
15054	*cspI*	Cold shock protein	0.52		
15707	*sspB*	Stringent starvation protein B	0.61		
16112	*Soda*	Superoxide dismutase, manganese	0.61	0.42±0.11*	
17062	*cutC*	Copper homeostasis protein	0.66		
17551		Peroxiredoxin	0.66		
**Regulator**					
14556	*ychA*	Putative regulator	0.60		
18914	*Dps*	DNA-binding ferritin-like protein	0.64	0.44±0.09*	
**Transporter**					
18318	*ybeX*	Mg^2+^ and Co^2+^ transporter CorC	0.60	0.61±0.08*	
19446		Putative ABC transporter	0.58		
**Metabolism**					
15181	*fbaA*	Fructose-bisphosphate aldolase, class II	0.66	0.21±0.05*	
16737	*fkpB*	FKBP-type peptidyl-prolyl cis-trans isomerase	0.65	0.69±0.12	
17825	*lysC*	Aspartokinase III, lysine sensitive	0.56		+
20228	*moaA*	Molybdenum cofactor biosynthesis protein	0.67		
20476	*Dcd*	2′-deoxycytidine 5′-triphosphate deaminase	0.65		
20585	*Pta*	Phosphate acetyltransferase	0.67 b		
20642	*adhE*	Acetaldehyde dehydrogenase and iron-dependent alcohol dehydrogenase	0.62		
**Unknown**					
14752		Unknown protein	0.64 b		
14753		Unknown protein	0.58 b		
14754		Unknown protein	0.65 b		
14913		Unknown protein	0.63		
15435		Unknown protein	0.63		
15908		Unknown protein	0.57	0.53±0.11*	
16290		Unknown protein	0.62		
**16827**		**Unknown protein**	**0.19**		
**17339**		**Unknown protein**	**0.17**	**0.53**±**0.10** *	
18387	*ybiJ*	putative exported protein	0.55	0.61±0.10*	
18337	*ybeD*	Unknown protein	0.60	0.39±0.07*	
18509		Unknown protein	0.65		
**19420**		**Unknown protein**	**0.50**		
19493		Unknown protein	0.64	0.58±0.19*	
19595		Unknown protein	0.58		
19558		Unknown protein	0.51		
**19953**		**Unknown protein**	**0.48** b		
19954		Unknown protein	0.58 b		
19955		Unknown protein	0.66 b		
20344		Unknown protein	0.67		
20437	*ygbE*	Membrane protein	0.63		
20587		Unknown protein	0.61 b	0.33±0.08*	
20588		Unknown protein	0.58 b		

a : Ratio for 3937 wild-type/*hrpL* mutant is the geomean of ten arrays (five slides, each slide has two arrays) from three biological replicates. HrpL down-regulated genes have the transcript abundances differing by 1.5-fold and SAM q-value less than 1% in minimal medium 6 h post inoculation at 28°C. Genes in bold font had at least a 2-fold change.

b : Several genes in one operon or overlapped genes are regulated.

c : The ratio of qRT-PCR from three replicates.

d : Hrp box was predicted with a hidden Markov model (HMM).

*: Significant difference determined by student t test (*p*<0.05).

Our microarray data correlated well with the qRT-PCR results. Out of the 16 HrpL-regulated genes examined by qRT-PCR, only gene ASAP15704 (Bcr/CflA subfamily drug resistance transporter) exhibited a microarray expression pattern that did not correspond to the qRT-PCR value. ASAP15704 appeared to be up-regulated in the microarray assay. However, the qRT-PCR result showed down-regulation without significant difference (Table 1). Due to extremely low expression of the dspA/E gene in the *hrpL* mutant, Ct value in the *hrpL* mutant background was high (greater than 35) compared to Ct value of 27 in wild-type 3937 background, which causes the large qPCR value variation although the qRT-PCR also indicated that *dspA/E* is upregulated by HrpL (Table 1, 2). However, even *dspA/E* was not included in the qRT-PCR and microarray correlation calculation, there is still reasonably high correlation (R^2^ = 0.63) between the microarray and qPCR results for the remaining 15 genes (Table 1, 2).

### Bioinformatics-based determination of HrpL-regulated genes

To further characterize the set of *D. dadantii* HrpL-regulated genes, a hidden Markov model (HMM) was used to analyze the *hrp* box consensus sequence of 3937. Using 69 *hrp* boxes from four species as training sets (Supplementary Table S2), we identified 73 genes with a potential *hrp* box, although only 11 genes with HMMER bit scores >8.5 were considered as reliable (Supplementary Table S3).

Among the HrpL up-regulated genes identified in the microarray assay, only *hrpA, hrpF, hrpJ, hrpK, hrpN*, and *hrpW* were found to contain a *hrp* box sequence in their promoter regions (bit score >8.5). Surprisingly, *dspA/E* was not included in this list of predicted *hrp* boxes, which may be due to the presence of a mis-predicted CDS in the source genome that would obscure identification of a *hrp* box (Supplementary Table S3).

### HrpL specifically binds to promoter regions of T3SS genes together with RNAP

HrpL is suggested to form a complex with core RNA polymerase (RNAP) and bind to the *hrp* box sequence in the promoter regions of T3SS genes of phytopathogens. However, few reports have demonstrated a direct interaction between HrpL and RNAP at specific regulatory regions for T3SS genes. In our microarray assay *hrpA* and *hrpN* were found to be up-regulated by HrpL (Table 1). In addition, the HMM analysis (Supplementary Table S3) identified putative *hrp* box sequences in the promoter regions of *hrpA* and *hrpN* (putative *hrp* box is underlined and listed in Supplementary Table S1).

**Figure 1:**
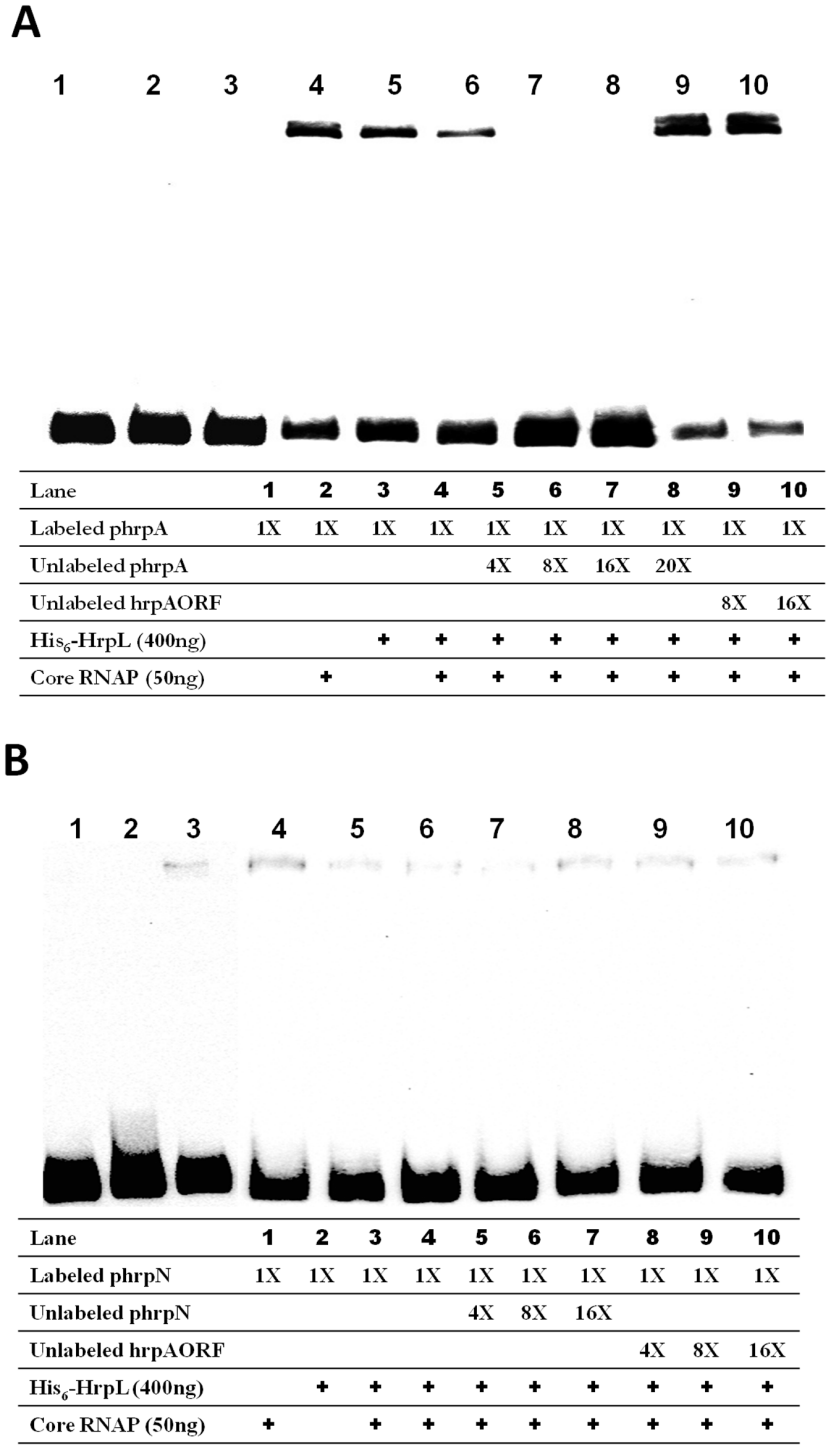
HrpL binds to *hrp* box specifically. *hrpA* or *hrpN* promoter regions containing the *hrp* box were amplified by PCR and labeled with digoxigenin (DIG) (Pierce, Rockford, IL). The core RNAP or His_6_-HrpL sigma factor alone were mixed with 200 fmol labeled DNA probe (1×) (Lane 1–2); or core RNAP and His_6_-HrpL sigma factor mixture together with 1× labeled DNA probe were mixed with same unlabelled DNA probe with various concentrations up to 4000 fmol (20×), or up to 3200 fmol (16×) of unlabeled internal fragment of the *hrpA* gene. **A**: Labeled and unlabeled *hrpA* promoter region containing *hrp* box and the control fragments for the *hrpA* ORF region without *hrp* box (hrpAORF) were used. **B**: Labeled and unlabelled *hrpN* promoter region containing *hrp* box and the control fragments for the *hrpA* ORF region without *hrp* box were used.

To study the interaction of the HrpL protein with the promoter regions of *hrpA* and *hrpN* in 3937, an electrophoretic mobility shift assay (EMSA) was employed. RNAP (Epicentre Technologies, Madison, WI) and the His_6_-tagged HrpL (His_6_-HrpL) were incubated together with digoxigenin (DIG) labeled DNA fragments of *hrpA* or *hrpN* promoter regions containing *hrp* box sequences. Neither the His_6_-HrpL nor RNAP alone showed detectable binding to the *hrp* box DNAs of *hrpA* and *hrpN* (Fig. 1). However, mobility of the DNA fragments was retarded when *hrp* box DNA of *hrpA* or *hrpN* was incubated with RNAP and His_6_-HrpL (Fig. 1), indicative of the requirement of RNAP in the binding of HrpL to these DNA fragments.

In addition, the specificity of RNAP-His_6_-HrpL binding to the promoter regions of *hrpA* and *hrpN* was demonstrated by a competition assay. Unlabeled DNA fragments of *hrpA* or *hrpN* promoter regions were able to compete with DIG-labeled *hrpA* or *hrpN* promoter regions to form RNAP-His_6_-HrpL -*hrpA* or RNAP-His_6_-HrpL –*hrpN* complexes (Fig. 1). Finally, when an internal DNA fragment of the *hrpA* or *hrpN* gene was used, it was unable to compete with the labeled *hrpA* promoter region (Fig. 1A) or labeled *hrpN* promoter region (Fig. 1B). These results are different from the competition effect observed with unlabeled promoter regions of T3SS genes (Fig. 1), and suggest specific binding between the RNAP-His_6_-HrpL complex and the promoter regions of *hrpA* and *hrpN* (Fig. 1).

### The T3SS effector DspA/E is translocated into the plant host through a functional T3SS

Among all of the HrpL up-regulated genes identified in the microarray study (Table 1) and HMM bioinformatics prediction (Supplementary Table S3), *dspA/E* is the only gene confirmed to encode a potential T3SS effector in 3937 [24]. In several phytopathogens, T3SS effectors function inside the host cytosol and interfere with host defenses by targeting the host cytoskeleton and defense system [25]. While several studies have demonstrated that the T3SS in *D. dadantii* plays a role in pathogenicity during the initial stages of infection [20], [21], [23], [26], [27], there has been no direct evidence for the translocation of effectors by this T3SS to the plant cytosol.

The Cya protein is a calmodulin-dependent adenylate cyclase that has been widely used as a reporter to study the translocation of proteins by bacterial pathogens [28]–[31]. Cya is active only upon delivery into the host cell cytosol, where it elevates cyclic AMP levels [32]. To investigate the translocation of DspA/E from 3937 into plant host cells, the Cya protein from *Bordetella pertussis* was used as a reporter [32]. Cya was fused to the C-terminal end of full-length DspA/E, or to the first 315 amino acids of the protein, resulting in DspE-Cya and DspE_315_-Cya fusion proteins, respectively (Supplementary Table 1). The corresponding plasmids were transformed into a 3937 *outC* mutant strain A1919, and an *outC*/*hrcV* double mutant strain Ech141. *outC* encodes an inner membrane protein of the type II secretion system (T2SS), which is responsible for the secretion of PCWDEs, including pectinases. Mutations in *outC* therefore prevent secretion of these enzymes and, in turn, reduce or eliminate plant cell maceration [33]. *hrcV* encodes a structural component of the T3SS, and a mutation in this gene is expected to prevent translocation of T3SS-dependent proteins [34].

An *outC* mutant strain A1919 and an *outC*/*hrcV* double mutant strain Ech141 carrying constructs that encode either full-length DspA/E (pDspE-Cya) or the first 315 amino acids of DspA/E (pDspE_315_-Cya) fused to Cya were infiltrated into *Nicotiana benthamiana*, and the concentration of cAMP (picomoles of cAMP per microgram of protein, ± standard errors) was determined 7 h post infiltration. Compared with the cAMP concentration (0.03±0.01) for *N. benthamiana* leaves infiltrated with the T2SS/T3SS double mutant strain Ech141 harboring pDspE-Cya, a significantly higher concentration of cAMP (0.97±0.04; *p* = 0.018, t-test) was observed in *N. benthamiana* leaves infiltrated with the T2SS mutant A1919 carrying pDspE-Cya (Table 3). Similarly, the concentration of cAMP (0.80±0.02) from *N. benthamiana* leaves infiltrated with A1919 (pDspE_315_-Cya) was significantly higher than the concentration (0.04±0.04; *p* = 0.003, t-test) from Ech141 (pDspE_315_-Cya). These results demonstrate that DspA/E of 3937 is translocated into the cytosol of *N. benthamiana* in a T3SS-dependent manner, and that the 315-amino-acid N-terminal fragment contains the translocation signal.

**Table 3:** Calmodulin-dependent adenylate cyclase activity of *Dickeya dadantii* strains, A1919 and Ech141, carrying DspE-Cya and DspE_315_-Cya hybrid protein constructs in *Nicotiana benthamiana*.

	DspE-Cya	DspE_315_-Cya
**A1919**	0.97±0.04	0.80±0.02
**Ech141**	0.03±0.01	0.04±0.04

An *outC* mutant strain A1919 and an *outC*/*hrcV* double mutant strain Ech141 carrying constructs that encoded either full-length DspE (pDspE-Cya) or the first 315 amino acids of DspE (pDspE_315_-Cya) fused to Cya were infiltrated into *N. benthamiana* at an OD_600_ of 0.3. cAMP production was assayed 7 h post infiltration as described [30]. cAMP was quantified in triplicate for each sample, cAMP levels are reported in picomoles of cAMP per micrograms of protein with standard errors. This experiment was performed twice and similar results were observed. Values are a representative of these two experiments. The *N. benthamiana* leaves infiltrated with buffer only demonstrated a cAMP level of 0.02±0.00.

As a negative control, the *N. benthamiana* leaves infiltrated with buffer only had a cAMP level of 0.02±0.00. In addition, Cya fusion proteins (p20514-Cya and p20422-Cya) were constructed with genes unrelated to T3SS (Supplementary Table S1). p20514-Cya and p20422-Cya contain full length genes that encode a putative cytoplasmic protein and the DNA-binding transcriptional regulatory protein FabR, respectively. No significant amount of cAMP was detected in *Nicotiana benthamiana* leaves infiltrated with the wild-type strain carrying p20514-Cya or p20422-Cya (0.01±0.00 and 0.01±0.01, respectively).

## Discussion

Many hypersensitive response and pathogenicity (Hrp) outer proteins (Hops) and effectors have been discovered in biotrophic phytopathogens, including *P. syringae* [5], [25], [35]–[38]. In this report, microarray analysis with the necrotrophic pathogen 3937 and its *hrpL* mutant demonstrated that the only known effector, *dspA/E,* is up-regulated by HrpL. Similarly, only a limited number of T3SS-related genes were predicted to contain a *hrp* box based on an HMM search using promoter regions of genes identified in the microarray as being up-regulated by HrpL (Supplementary Table S3). These genes include *hrpK, hrpN, hrpF* in the *hrpF* operon, *hrpJ* in the *hrpJ* operon, *hrpW* in the *dspA/E* operon, and *hrpA* in the *hrpA* operon (Supplementary Table S3; Supplementary Fig. S2). When applied to all 3937 intergenic regions, the HMM predicts only five additional *hrp* boxes with confidence (Supplementary Table S3; Supplementary Fig. S2). Although the microarray assay results demonstrated more HrpL-downregulated genes than those upregulated by HrpL (Table 1, 2; Supplementary Fig. S2), none of the HrpL-downregulated genes had a *hrp* box HMM prediction with a high confidence level (Table 2; Supplementary Table S4; Supplementary Fig. S2H-I). Nearly half of the HrpL-upregulated genes were annotated as T3SS associated genes in the 3937 genome and about half of the highly confident HMM predicted genes were also identified by microarray (Table 1; Supplementary Table S4; Supplementary Fig. S2E-F). The correlation among T3SS genes identified by genome annotation, microarray, and HMM prediction indicates that HrpL majorly upregulates T3SS genes, and the genes down-regulated by HrpL may be due to indirect regulation (Table 1, 2; Supplementary Table S4; Supplementary Fig. S2).

In addition, ten T3SS associated genes in the 3937 genome were determined to be up-regulated by microarray with an arbitrary cut-off value of 1.5-fold change. The number of T3SS genes identified by microarray drops significantly from ten to six HrpL-upregulated genes if the array cut-off value is set at a 2-fold difference (Table 1, Supplementary Table S4; Supplementary Fig. S2). Although there may be some false-positive HrpL-regulated genes identified using a 1.5-fold cut-off value, the false-negative rate is likely to increase if we apply a 2-fold change cut-off value in this study. In contrast, the number of genes in common among the set of T3SS associated genes in the 3937 genome and the HMM predicted genes did not change when the confidence level of the HMM prediction increased (Table 1, 2; Supplementary Table S4; Supplementary Fig. S2). Therefore, we used a 1.5-fold cut-off value for microarray analysis and a confidence level (HMM score) of 8.5.

If it is to be assumed that all T3SS effectors are preceded by a HrpL binding site, then this would imply that 3937 contains very few potential effectors. An alternative explanation is that some lower confidence *hrp* box predictions are in fact valid HrpL binding sites. Although the reason for 3937 and related necrorophic pathogens to have so few predicted effectors compared to biotrophic pathogens is unknown, and with the proviso that more may yet be discovered, it is possible that this low number is associated with the different modes of infection employed by necrotrophic and biotrophic plant pathogens. In biotrophic phytopathogens, the T3SS appears to be the major virulence determinant and is required for the bacterium to disarm the host defense responses during bacterial invasion.

In *D. dadantii*, however, the pectinases secreted from the T2SS play a more important role in pathogenesis [17], [39]. Rather than eliminating pathogenicity, most T3SS mutants of *D. dadantii* show a delayed maceration of plant tissues [21], [23], [27]. This suggests that the T3SS of *D. dadantii* functions to promote the initial establishment of the bacterium in host plants during the early stages of infection. This is supported by evidence that the T3SS genes of *D. dadantii* are expressed during the early stages of infection [9], together with a delay in the expression of pectate lyases [9], [40]. However, this hypothesis remains to be tested for *D. dadantii* as recent transcriptome data from the related pathogen, *Pectobacterium atrosepticum*, suggest that the T3SS may be under quorum sensing control and, therefore, coordinately expressed later in infection together with the PCWDEs [41]. The finding that the *hrp* box sequence was mainly found in the T3SS genes identified in our microarray assay, suggests that many of the other genes identified in this array may be indirectly regulated by HrpL. Finally, although some T3SS genes e.g. *dspF* (ASAP19013) identified in the 3937 genome sequence (https://asap.ahabs.wisc.edu/asap/ASAP1.htm) were differentially expressed, they were below the cut-off value of 1.5-fold change and thus not included in Table 1.

HrpL is an RpoN-dependent ECF family sigma factor that regulates the expression of many T3SS genes, including those encoding effectors and assembly of the T3SS apparatus. It is suggested that HrpL associates with the core RNA polymerase and binds to the *hrp* box consensus sequence in the promoter regions of T3SS genes to initiate transcription [25]. In this study, a direct interaction between the HrpL protein and the promoter regions of *hrpA* (Fig. 1A) and *hrpN* (Fig. 1B) in 3937 was demonstrated by EMSA. The competition assays indicated that the binding between HrpL and the promoter regions of *hrpA* and *hrpN* was specific, suggesting that the HrpL protein directly regulates the transcriptional expression of these genes. In addition, the binding affinity of HrpL for the *hrp* box of different T3SS genes is different. For example, the binding of HrpL to the *hrp* box of *hrpA* (Fig. 1A) seems to be stronger than to that of *hrpN* (Fig. 1B). When added to a mixture of core RNAP and His_6_-HrpL sigma factor, the band intensity (Fig. 1A) was stronger using a *hrpA* labeled DNA probe than when the same amount (200 fmol) of *hrpN* labeled DNA probe was used (Fig. 1B). Interestingly, we observed a correlation between the binding affinity of HrpL and the expression level of HrpL-induced genes: the relatively higher affinity of HrpL for the *hrpA* promoter (Fig. 1A) renders *hrpA* expression over four-fold higher than that of *hrpN* based on the microarray data (Table 1).

The calmodulin-dependent Cya protein is not secreted or translocated by the T3SS, [32], and is inactive inside the bacterial cytosol due to the absence of calmodulin. These properties make Cya a good reporter for T3SS translocation research in bacterial pathogens [28]–[31]. To avoid false positive results during our analysis of T3SS translocation as a consequence of plant tissue maceration by pectinases secreted from 3937, an *outC* mutant was used. This mutant is unable to secrete pectinases through the T2SS [33]. *hrcV* encodes an inner membrane structural component of the T3SS apparatus and is essential for secretion of effectors [34]. We used single mutants of either *outC*, or *hrcV* and a double mutant *outC/hrcV* to demonstrate that DspA/E from 3937 is delivered into the plant cytosol in a T3SS-dependent manner. In addition, a plasmid containing only the first 315 amino acids of DspA/E was used to show that the T3SS secretion signal is located in the first one-fifth of the N-terminus of the DspA/E protein. This latter result is consistent with other reports, where T3SS secretion signals have been found within the first 20–150 amino acids of T3SS substrates [42]–[45].

In summary, using microarrays, *hrp* binding site prediction, EMSA, and Cya translocation assays, we provide the genome-wide study of HrpL-regulated genes in a necrotrophic phytopathogen. Specific binding between the alternative sigma factor HrpL and promoter regions of T3SS genes *hrpA* and *hrpN* was observed. Finally, in sharp contrast to the biotrophic pathogen *P. syringae* pv. *tomato*, only a single effector (DpsA/E) has so far been identified in 3937, which we have shown to be translocated by the 3937 T3SS into the plant cytosol, where it functions in virulence. The T3SS in this necrotrophic pathogen may thus play a minor role during infection compared to biotrophic pathogens, instead relying more on its ability to physically attack the plant through the production of PCWDEs.

## Materials and Methods

### Bacterial strains and plasmids

Bacterial strains, plasmids, and primers used in this study are listed in Supplementary Table S1. Wild-type 3937 and its mutant strains were stored at –80°C in 15% glycerol and grown on Luria-Bertani (LB) agar or T3SS-inducing minimal medium (MM) [9]. Plasmids were isolated from *Escherichia coli* DH5α using QIAprep Spin Miniprep Kit (Qiagen, Valencia, CA). Plasmids were introduced into bacterial strains by electroporation using a Gene Pulser Electroporation system (Bio-Rad, Hercules, CA). Antibiotics were used at the following concentrations: ampicillin, 100 µg/mL; chloramphenicol, 50 µg/mL; kanamycin, 50 µg/mL; spectinomycin, 50 µg/mL. The *hrpL* mutant WPP96 was used in this microarray assay, and this mutant was complemented by plasmid *phrpL* in a previous study [12].

### Construction of mutants

To construct the *outC/hrcV* double mutant, a 2119-bp *BamH*I-*Xho*I fragment containing the full *hrcV* ORF was amplified using primers hrcV-F and hrcV-R. This fragment was digested by *Pst*I and re-ligated following removal of a 417-bp internal region of *hrcV*. The recombinant fragment was inserted into pWM91 at the *BamH*I and *Xho*I sites, resulting in the plasmid pWM91ΔhrcV (Supplementary Table S1). The plasmid was introduced into the *outC* mutant strain A1919, and the *outC/hrcV* double mutant was generated by homologous recombination and verified by DNA sequencing analysis.

### RNA isolation and preparation of labeled cDNA

Wild-type *D. dadantii* 3937 and WPP96 were grown in T3SS-inducing minimal medium for 6 h, and total RNA was isolated using a Bio-Rad Aurum Total RNA Mini Kit as described by the manufacturer (Bio-Rad, Hercules, CA). RNA was quantified using a ND-100 spectrophotometer (NanoDrop Technologies, Wilmington, DE), and the quality was assessed on an agarose gel.

RNA was labeled as previously described [46] with minor modifications. Briefly, 12 µg of total RNA was labeled in a 45-µL labeling reaction with 10 µL of a combination of 1 µL of spike RNA mix (GE Healthcare, Piscataway, NJ), 1.8 µL of 25× amino allyl deoxynucleoside triphosphate (dNTP) mixture [5 µL each of dATP, dCTP, dGTP (100 mM), 2 µL dTTP (100 mM), 6 µL aminoallyl dUTP, and 17 µL SDW], 4.5 µL of dithiothreitol (0.1 M), 2 µL of 1 mg/mL Oligo dT18 primers (Ambion, Austin, TX), and 2 µL of reverse transcriptase in reaction buffer (Ambion, Austin, TX). Primers and RNA were heated to 70°C for 10 min and then cooled on ice following incubation for 2 h at 42°C. The remaining RNA was denatured with 15 µL of 1 M NaOH and 15 µL of 0.5 M EDTA (pH 8.0) at 65°C for 10 min following neutralization with 15 µL of 1 M HCl.

Purification of cDNA was performed as described by the supplier (Qiagen, Valencia, CA). One microliter of the appropriate Cy dye ester suspended in dimethyl sulfoxide (GE Healthcare, Piscataway, NJ) and 2.0 µL of 1 M sodium carbonate were added to the purified cDNA in amber tubes and incubated for 1 h at room temperature in the dark. To the labeled cDNA, 3.0 µL of 4.0 M hydroxylamine hydrochloride was added and incubation was extended for an additional 30 min in the dark. The labeled targets were combined and mixed with distilled water and further purified with a QIAGEN MiniElute column (Qiagen, Valencia, CA). The labeling efficiency was estimated with 1 µL of the cDNA using a NanoDrop ND-100 spectrophotometer.

### Hybridization to DNA microarrays and image analysis

*D. dadantii* microarrays (AMADID No. 012716) were developed at the Scottish Crop Research Institute (SCRI), Dundee, UK, through Agilent Technologies, Inc. (Santa Clara, CA) based on the genome sequence of strain 3937. The microarray experiment was carried out as described [46] with minor modifications. Briefly, labeled cDNA was made up to a volume of 80 µL with distilled water, denatured at 98°C for 3 min, and then mixed with 25 µL of control target and 105 µL of 2× hybridization buffer (Agilent, Santa Clara, CA). The hybridization, wash, and dry were carried out in a Lucidea™ Slidepro hybridization chamber (GE Healthcare, Piscataway, NJ). After hybridization for 17 h at 60°C, the arrays were washed for 1 min each with wash solution I (6× SSPE [1× SSPE is 0.18 M NaCl, 10 mM NaH_2_PO_4_, and 1 mM EDTA, pH 7.7], 0.005% *N*-lauroylsarcosine), followed by wash solution II (0.06× SSPE, 0.005% *N*-lauroylsarcosine).

Arrays were scanned using a GenePix 4200A scanner (Molecular Devices, Sunnyvale, CA) with appropriate exposure settings for Cy3 (595 nm) and Cy5 (685 nm) at a 10-µm resolution, generating separate TIFF images. Signal intensity and ratios were generated using GenePix Pro 6 software (Molecular Devices, Sunnyvale, CA). Images were imported and aligned with clone position information (Agilent GAL file) using automated and manual grid alignment features. Median spot and individual median background (annulus setting) intensity values were extracted for each wavelength and imported into analysis software.

### Microarray normalization and analysis

Microarray data with intensities reproducibly higher than background from 10 array results of three biological replicate experiments were selected for analysis. Following local background subtraction, the signal for each spot was normalized based on the geomean value of the median intensity of all the spots for each array as described [47]. Each ratio (*hrpL* mutant/3937 wild type) was converted to its log2 value for Significance Analysis of Microarrays (SAM) software analysis [48]. Genes showing differential expression in the microarray assays were determined by setting the number of falsely called genes to less than one for the significant gene list output. Significant genes compared to the geometric mean of the ratios from ten arrays were considered as HrpL-up-regulated if their level of transcription in the *hrpL* mutant WPP96 was no more than two-thirds of that observed in the corresponding 3937 wild type. Genes were considered HrpL-down-regulated if their level of transcription was at least 1.5-fold higher in the *hrpL* mutant WPP96 than that of wild-type 3937. The microarray data have been deposited into the GEO database with an accession number of GSE17185.

### Real-time qRT-PCR

For independent confirmation of gene expression results obtained with oligonucleotide microarrays, 3937 and the *hrpL* mutant WPP96 were grown in T3SS-inducing minimal medium. Total RNA from the bacteria was isolated using the TRI reagent method (Sigma, St. Louis, MO) and treated with the Turbo DNA-free DNase kit (Ambion, Austin, TX). These RNA preparations were independent from those used for microarray hybridizations. An iScript cDNA Synthesis Kit (Bio-Rad, Hercules, CA) was used to synthesize cDNA from 0.5 µg of treated total RNA. The Real Master Mix (Eppendorf, Westbury, NY) was used for a real time PCR reaction to quantify the cDNA level of target genes in different samples. The *rplU* gene was used as the endogenous control for data analysis [10]. The primer pairs used in this study are listed in Supplementary Table S1. Reactions were run and data collected using the Opticon™ 2 system (Bio-Rad, Hercules, CA). All PCR experiments were performed in triplicate, and standard deviations were calculated.

### HrpL binding site prediction by HMM

A training set of 69 HrpL binding sites extending from the −36 to −7 positions was compiled from literature sources (supplementary Table S2). These sequences were aligned using Jalview [49] and the resulting alignment used as input to construct hidden Markov models (HMMs) with the *hmmbuild* and *hmmcalibrate* packages of HMMer (http://hmmer.janelia.org/). The HMMer package *hmmsearch* was used to query test data sets and the target genome. Parameters for model construction (background and null models, HMMer version) were optimized by ten-fold cross-validation on the 69 sequence dataset. An optimal model was indicated, with a bit-score classifier threshold of 8.5. This model and threshold were applied to intergenic regions derived from the 3937 genome sequence obtained from ASAP (http://asap.ahabs.wisc.edu/software/asap/) to predict candidate HrpL-binding sites.

### Expression and purification of His_6_-HrpL protein

For HrpL protein expression, *hrpL* was amplified by PCR using Phusion high-fidelity DNA polymerase (New England Biolabs, Ipswich, MA) from 3937 chromosomal DNA with primer pairs hrpL-PF/hrpL-PR (Supplementary Table S1). PCR fragments were gel purified and ligated into the pENTR/SD/D-TOPO vector (Invitrogen, Carlsbad, CA). The resulting pENTR-HrpL constructs were then digested with *Nde*I and *Hind*III and ligated into *Nde*I/*Hind*III linearized pET-28a(+) vectors (Novagen, Madison, WI) to produce plasmid phrpL-28a.

An overnight LB culture of *E. coli* BL21 (DE3) containing His_6_-HrpL encoding plasmids phrpL-28a was subcultured into 100 mL of LB broth containing kanamycin. Bacterial cells were grown at 37°C with shaking until an OD_600nm_ of 0.5 was reached. Following addition of IPTG (isopropyl-beta-thio galactopyranoside) to a final concentration of 1 mM, the culture was incubated for an additional 4 h to induce the expression of the *his_6_-hrpL* gene. The bacterial cells were harvested by centrifugation at 22,000×*g*, resuspended in 20 mL of water, and lysed by freezing and thawing. The lysate was sonicated on ice and centrifuged. The insoluble pellet was washed twice with a washing buffer (25% [wt/vol] sucrose, 5 mM EDTA, 1% [vol/vol] Triton X-100 in phosphate-buffered saline) and suspended in 5 mL of denaturing solution (6 M guanidinium hydrochloride, 0.1 M Tris-HCl buffer [pH 8.0], 1 mM EDTA [pH 8.0], 0.03 M β-mercaptoethanol). Protein renaturation was initiated by 20-fold dilution in 0.2 M ammonium acetate (pH 8.5). After 48 h of incubation at room temperature, the insoluble protein was removed by filtration through a 1-mm Whatman filter paper and the soluble protein was precipitated with 55% saturated ammonium sulfate overnight. The precipitate was collected by filtration through a 0.45 µm Fisherbrand Nylon filter and solubilized in 2 mL of 10 mM Tris-HCl (pH 8.0). The concentration of purified HrpL protein was measured by the BCA protein assay kit (Bio-Rad, Hercules, CA).

The purified His_6_-tagged HrpL protein was confirmed by Western Blot using mouse anti-His antibody (GE Healthcare, Piscataway, NJ) as a primary antibody and goat anti-mouse Ig(H+L)-HRP (Southern Biotech, Birmingham, AL) as a secondary antibody followed by detection with an Immun-Star™ HRP Substrate Kit (Bio-Rad, Hercules, CA).

### Electrophoretic mobility shift assay

Primers were designed to amplify fragments containing the *hrp* box in promoter regions of *hrpA* and *hrpN*. PCR products were labeled with DIG using the Biotin 3′ end DNA labeling Kit (Pierce, Rockford, IL). In EMSA, a core RNAP (50 ng) (Epicentre Technologies, Madison, WI) was incubated on ice for 20 min with 400 ng of purified His_6_-HrpL protein. The core RNAP and His_6_-HrpL sigma factor mixture was added into eppendorf tubes containing the DNA probe (200 fmol), 1 µg Poly (dI·dC), DNA binding buffer, 50% Glyerol, 100 mM MgCl_2_, and 1% NP-40 (Pierce, Rockford, IL) to give a final volume of 20 µL. Following incubation at 28°C for 20 min, the samples were loaded onto a 6% native polyacrylamide gel and eletrophoresed in 0.5× TBE. The DNA probes were transferred to an N-positive nylon membrane and detected according to the manufacturer's protocol of chemiluminescent nucleic acid detection module (Pierce, Rockford, IL)

### Construction of *cya* fusion plasmid

The *cya* fusions were constructed using the Gateway cloning technology (Invitrogen, Carlsbad, CA) as described [30]. In brief, the appropriate PCR products were cloned into the pENTR/SD/D-TOPO vector (Invitrogen, Carlsbad, CA) to create the entry clone. The PCR products were then transferred by recombination into the destination vector, pCPP3234, which contains the *cya* gene without the start codon, to create an in-frame fusion of the target gene *dspA/E* and *cya* generating the constructs pDspE-Cya and pDspE_315_-Cya.

### Quantification of cAMP *in planta*

Mutants derived from 3937 were infiltrated into leaves of *N. benthamiana* at a cell density of 0.3 (OD_600nm_). Leaf disks were harvested from the infiltrated areas 7 h post infiltration with a 1-cm-diameter cork borer and frozen in liquid nitrogen. Frozen disks were prepared for cAMP quantification as described [30]. The cAMP content in samples was determined with the Correlate-EIA Direct Cyclic AMP Enzyme Immunoassay Kit (Assay Designs, Inc., Ann Arbor, MI), following the manufacturer's instructions. Protein concentration in the samples was determined with Bio-Rad protein assay kit (Bio-Rad, Hercules, CA). cAMP was quantified in triplicate for each sample, and reported in picomoles of cAMP per micrograms of protein with standard errors. This experiment was performed twice, and similar results were observed. Values are a representative of these two experiments.

## Supporting Information

Table S1 Strains, plasmids, and DNA primers used in this study.
(0.11 MB DOC)

Table S2 The source of each of the hrp boxes comprising the hrp box training set. This data set is made up of sixty-nine sequences from four distinct species which share a recent common ancestor.
(0.19 MB DOC)

Table S3 HMM predictions of the locations of hrp boxes having bit score greater than the optimal prediction threshold 8.5. Columns represent: Feature ID of Dickeya dadantii 3937 ASAP accession ID of version v6b; HMMer bit score for the prediction; distance between prediction and the closest downstream gene; name of closest downstream gene; annotated product of closest downstream gene; orientation of closest downstream gene; orientation of HMMer prediction.
(0.13 MB DOC)

Table S4 Genes associated with type III secretion pathway in 3937.
(0.12 MB DOC)

Figure S1 Functional categories of HrpL-regulated genes by comparing the transcriptome profiles of Dickeya dadantii 3937 wild-type and its hrpL mutant in T3SS inducing minimal medium after 6-h post inoculation at 28oC.
(0.11 MB DOC)

Figure S2 Correlation among the T3SS genes in 3937 genome, HMM prediction, and microarray results. Genome: Dickeya dadantii 3937 T3SS gene ASAP accession ID of version v6b based on the annotation in ASAP website (Supplemental Table 4). Array_All: all the HrpL-regulated genes from microarray experiment with a cut-off value of 1.5-fold (Table 1, 2). Array_UP: all the HrpL-upregulated genes (Table 1). Array_DOWN: all the HrpL-downregulated genes (Table 2). Array_2Fold: all the HrpL-regulated genes with cut-off value of 2-fold changes (Bold ones in Table 1, 2). Array_UP_2: HrpL-upregulated genes with at least 2-fold changes (Bold ones in Table 1). Array_DOWN_2: HrpL-downregulated genes with at least 2-fold changes (Bold ones in Table 2). HMM_All: all the genes predicted by HMM (Supplemental Table 3). HMM_SIG: HMM predicted genes with HMM score greater than 8.5 (Bold ones in Supplemental Table 3).
(0.48 MB DOC)
