# Supplementary material for: Genome-Wide Identification of HrpL-Regulated Genes in the Necrotrophic Phytopathogen Dickeya dadantii 3937
Source: PLoS One. 2010 Oct 19;5(10):e13472. doi: 10.1371/journal.pone.0013472 (PMC2957411; doi:10.1371/journal.pone.0013472)
Supplement: Table S1 — Strains, plasmids, and DNA primers used in this study. (0.11 MB DOC) [file pone.0013472.s001.doc]

| **Strains, plasmids, and primers** | **Characters or sequences (5**' **to 3**'**)^a^** | **Reference or source** |
| --- | --- | --- |
| **Strains** |  |  |
| ***E. coli*** |  |  |
| *E. coli* DH5α | F^-^ 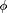80*lacZ*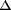*M15* 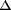*lacZYA-argF*)*U169 deoR recA1 endA1 hsdR17 phoA supE44 thi-1 gyrA96 relA1* 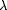^-^ | Invitrogen, CA |
| *E. coli* TOP10 | F^-^ *mcrA* 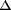*mrr-hsdRMS-mcrBC*) 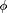80*lacZ*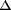*M15* 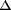*lacX74 deoR recA1 araD139* 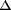(*ara-leu*)*7679 galU galK rpsL endA1 nupG* | Invitrogen, CA |
| *E. coli* BL21 (DE3) | F^-^*,ompT, hsdS_B_*(r_B-_m_B-_)*,dcm, gal, tonA* | Invitrogen, CA |
| ***Dickeya dadantii*** |  |  |
| 3937 | wild type, *Saintpaulia* (African violet) isolate | Hugouvieux-Cotte-Pattat, N. |
| WPP96 | Δ*hrpL*_(Δ1-185aa)_:: *aadA*; Sp^R^/Sm^R^ | [12] |
| A1919 | *lmrT^c^ lacZ2 outC::uidA*-Km^R^ | Guy Condemine |
| Ech141 | markerless *hrcV* deletion mutant of A1919 | This work |
| A1919 (pDspE-Cya) | A1919 containing pDspE-Cya; Km^R^ Sp^R^/Sm^R^ | This work |
| A1919 (pDspE_315_-Cya) | A1919 containing pDspE_315_-Cya; Km^R^ Sp^R^/Sm^R^ | This work |
| Ech141 (pDspE-Cya) | Ech141 containing pDspE-Cya; Km^R^ Sp^R^/Sm^R^ | This work |
| Ech141 (pDspE_315_-Cya) | Ech141 containing pDspE_315_-Cya; Km^R^ Sp^R^/Sm^R^ | This work |
| 3937 (p20514-Cya) | 3937 containing p20514-Cya; Sp^R^/Sm^R^ | This work |
| 3937 (p20422-Cya) | 3937 containing p20422-Cya; Sp^R^/Sm^R^ | This work |
| **Plasmids** |  |  |
| pCR2.1-TOPO | PCR cloning vector, Ap^R^ Km^R^ | Invitrogen, CA |
| pENTR/SD/D-TOPO | Gateway system donor vector, Km^R^ | Invitrogen, CA |
| pET-28a(+) | His_6_-tagged protein expression vector, Km^R^ | Novagen, WI |
| pCPP3234 | Gateway destination pVLT35 derivative containing the *cyaA* gene for C-terminal fusions , Sp^R^/Sm^R^Cm^R^ | [30] |
| phrpL-28a | pET-28a(+) derivative with PCR fragment containing *hrpL*, Km^R^ | This work |
| pENTR-dspE | pENTR/SD/D-TOPO containing PCR fragment encoding full length DspE except the stop codon, Km^R^ | This work |
| pENTR-dspE_315_ | pENTR/SD/D-TOPO containing PCR fragment encoding the first 315 amino acids of DspE , Km^R^ | This work |
| pDspE-Cya | pCPP3234 derivative obtained by recombination with pENTR-dspE, Sp^R^/Sm^R^ | This work |
| pDspE_315_-Cya | pCPP3234 derivative obtained by recombination with pENTR-dspE_315_, Sp^R^/Sm^R^ | This work |
| p20514-Cya | pCPP3234 derivative containing full length of gene with ASAP number ABF-0020514, which encodes a putative cytoplasmic protein, Sp^R^/Sm^R^ | This work |
| p20422-Cya | pCPP3234 derivative containing full length of gene with ASAP number ABF-0020422, which encode a DNA-binding transcriptional regulatory protein FabR, Sp^R^/Sm^R^ | This work |
| pWM91ΔhrcV | 1.7 kb fragment containing Δ*hrcV* in pWM91 | This work |
| **Primers** |  |  |
| *dspE_F* | CACCATGTTGGGCAATATCAATCATATTC | This work |
| *dspE_R* | CTGTTTCGCCAGCGTGATG | This work |
| *dspE315_R* | GTTGTCCTCTTTTTCGGTCA | This work |
| *hrcV-F* | AAAGGATCCCTGTGCTGATCGCCTGGCTTA | This work |
| *hrcV-R* | AGGCTCGAGTATGCGTTAAAGGGGTCAACC | This work |
| *Km FRT-F(EcoRI)* | AGAGAATTCGTGTAGGCTGGAGCTGCTT | This work |
| *Km FRT-R(EcoRI)* | AAAGAATTCATGGGAATTAGCCATGGTCC | This work |
| *hrpA_GS_F* | GGCTGTATCCTGTTATCACC | This work |
| *hrpA_GS_R* | GATTTCGCTATGAACTTTGCG | This work |
| *hrpN_GS_F* | CGGTTTCTTTTTTTGCGGCG | This work |
| *hrpN_GS_R* | AAGTATCTGCAACGGATGCC | This work |
| *hrpAORF-F* | GCAGTCTCTGGACAAAACCA | This work |
| *hrpAORF-R* | CTTGGTGGCTGAGTCCATCT | This work |
| *hrpL_PF* | CACCCATATGGAAACGATTACACTGAAAC | This work |
| *hrpL_PR* | AAGCTTTGCATCAACAGCCTGGC | This work |
| *rplU_RTF* | GCGGCAAAATCAAGGCTGAAGTCG | [10] |
| *rplU_RTR* | CGGTGGCCAGCCTGCTTACGGTAG | [10] |
| *15181F (fbaA)* | AGA AAT TCA ACC TGC CGC ACA ACA | This work |
| *15181R* | TCATCTTCACCACGCCGTAGCTG | This work |
| *15704F (unknown)* | CCTGATCTTACTGCTGGCGTTGCT | This work |
| *15704R* | GCTGCACCTTGCCCGATGAA | This work |
| *15908F (unknown)* | TCGTCCCTCTATCCGCCGTGG | This work |
| *15908R* | CGGACGTTAACACAGCGGCATCAT | This work |
| *16112F (soda)* | CGCATTCAAGGAAAAATTCGAGCA | This work |
| *16112R* | CTGGTTGGGGGTGGAAACGAC | This work |
| *16737F (fkpB)* | TTCTTCCTGCGCCGCGACTT | This work |
| *16737R* | GGTTGAAATCCACCGTCACCGACT | This work |
| *17339F (unknown)* | CTGATGATGGAACCGATGCGTT | This work |
| *17339R* | CATCACACAAAACCTCAACCACGA | This work |
| *17589F (cyoE)* | CGTTACTGTACCTCGCGGCAAATC | This work |
| *17589R* | CGCCTGACAGACTGCCAATCAAC | This work |
| *18318F (ybeX)* | CGCCCTTACGCCTATCGAGGACTT | This work |
| *18318R* | GCAGGTGACCGAAAGATTGCATCA | This work |
| *18337F (ybeD)* | TGCTCCCGGCGACTATAACCC | This work |
| *18337R* | CCCAGTTCTTCGTACAGCGTTTCC | This work |
| *18387F (ybiJ)* | AAACCATCGCTATCGCCGCTACC | This work |
| *18387R* | ACGGAACCGATTTTGTCCAACTGC | This work |
| *18914F (dps)* | TTCCGCACCTCGCTGATTACCCA | This work |
| *18914R* | CGGGTAGGGCTTCAGTGCGGTTTT | This work |
| *19004F (hrpK)* | CTGTTGGAACATGCGGACGTGA | This work |
| *19004R* | GCGGCGGCATCGTGAAAC | This work |
| *19006F (orfC)* | TGGTGACCCGGCGTCTCAA | This work |
| *19006R* | AAATCGGCTTCCAGTGAATCGACA | This work |
| *19012F (dspA/E)* | GATGGCGGAGCTGAAATCGTTC | This work |
| *19012R* | CCTTGCCGGACCGCTTATCATT | This work |
| *19493F (unknown)* | GTGGCTGGTGCATCCGCGTTTAGT | This work |
| *19493R* | GGTTGCGGTGGAGCTGTTCGTG | This work |
| *20587F (unknown)* | TCTGCTGCCGCCGTTTTTGTC | This work |
| *20587R* | TGGCCGGGTGATATTTCCAGACAT | This work |
| **DNA Fragments** |  |  |
| *hrpA hrp* box promoter | 5'-CGGTTTCTTTTTTTGCGGCGAACCGGCGACGGCAG**GGAACC**GTTTCACCGCTGGCGT**CACTCA**TTAACCAGTATCCATCATGATGCCCGCATCGGGATCGGAGCGGGCATCCGTTGCAGATACTT-3' | For Mobility-shift Assay |
| *hrpN hrp* box promoter | 5'-GGCTGTATCCTGTTATCACCACCAGGGGAAAAAAGTGAATATCTTTC**GGAACC**ACCTCGCATTATCTC**CTACTTA**ATCTATGAACACGATGACTGTGTAAGTCGCAAAGTTCATAGCGAAATC-3' | For Mobility-shift Assay |

^a^Ap^R^, ampicillin resistance; Cm^R^, chloramphenicol resistance; Km^R^, kanamycin resistance; Sp^R^, spectinomycin resistance; Sm^R^, streptomycin resistance. Restriction enzyme sites are underlined. *hrp* box is bold and underlined. The lengths of PCR products used for EMSA studies for primer sets *hrpA_GS_F/R, hrpN_GS_F/R,* and *hrpAORF_F/R* are 123, 125, and 121-bp respectively.
