# Supplementary material for: Genome-Wide Identification of HrpL-Regulated Genes in the Necrotrophic Phytopathogen Dickeya dadantii 3937
Source: PLoS One. 2010 Oct 19;5(10):e13472. doi: 10.1371/journal.pone.0013472 (PMC2957411; doi:10.1371/journal.pone.0013472)
Supplement: Table S2 — The source of each of the hrp boxes comprising the hrp box training set. This data set is made up of sixty-nine sequences from four distinct species which share a recent common ancestor. (0.19 MB DOC) [file pone.0013472.s002.doc]

| **Number** | **Tag** | **Acc. number** | ***Organism*** | **Pathovar** | **Strain** | ***Gene/Operon*** | **Training sequence (1-34 nuceotides)** | **Citation (Note)** |
| --- | --- | --- | --- | --- | --- | --- | --- | --- |
| ***Erwinia amylovora*** | | | | | | | | |
| **1** | Eam1 | Y13831 | *E. amylovora* | − | CFBP1430 | *dspA* | GGGAACCGTTTGC-AGA-GAATTGCAACATAAAA- | [50] |
| **2** | Eam2 | AJ698952 | *E. amylovora* | − | PD2915 | *hrpW* | CGGAACCCTGTCAACGCCAT--GCCCACTCAATT- | [51] |
| **3** | Eam3 | L25828 | *E. amylovora* | − | − | *hrpJ* | GGGAACCGAT-GC--GT-CAATCGCACCACACAA- | [52] |
| **4** | Eam4 | L25828 | *E. amylovora* | − | − | *ORF12* | CGGAACTATTACC-TGCCGTTC-GCCACCTATTC- | [52] |
| **5** | Eam5 | U94513 | *E. amylovora* | − | − | *hrpW* | CGGAACCCTGTCAACGCCAA--ACCCACTCAATT- | [53, 54] |
| **6** | Eam6 | U97504 | *E. amylovora* | − | − | *dspE* | GGGAACCGGTTGC-AGA-GAATTGCAACATAAAA- | [25] |
| **7** | Eam7 | U56662 | *E. amylovora* | − | Ea321 | *hrpA* | GGGAACCGATCGAAACTGC--CCGCCACTTAATT- | [55] |
| **8** | Eam8 | U56662 | *E. amylovora* | − | Ea321 | *hrpF/hrpC* | CGGAACTCCGCCACGCCCGA-ACCCCACTCAAAG- | [55] |
| **9** | Eam9 | U56662 | *E. amylovora* | − | Ea321 | *hrpF/hrpC* | CGAACCCCACTCAAAGACAG-GACCCACTCAATG- | [55] |
| **10** | Eam10 | U56662 | *E. amylovora* | − | Ea321 | *hrpN* | CGGAACCAGAGCG-GAATAA-CCAGCACTCAATA- | [56] |
| ***Erwinia chrysanthemi*** | | | | | | | | |
| **11** | Ech1 | AF501263 | *E. chrysanthemi* | − | EC16 | *hrpF* | TGGAACCGCCG-C-CACTCCCCGGCCACACAACT- | [57] |
| **12** | Ech2 | AF501263 | *E. chrysanthemi* | − | EC16 | *hrpN* | AGGAACCGTTTCA--CCGTCGGCGTCACTCAGTA- | [57] |
| ***Pectobacterium atrosepticum*** | | | | | | | | |
| **13** | Pba1 | − | *P. atrosepticum* | − | SCRI1043 | *hrpJ* | GGGAACCCATCC--TTTTCTGCGTCCACACAGCA- | [34] |
| **14** | Pba2 | − | *P. atrosepticum* | − | SCRI1043 | *hrpA* | GGGAACTCCTCCA-GCCCGA-TCTCTACTTAATG- | [34] |
| **15** | Pba3 | − | *P. atrosepticum* | − | SCRI1043 | *hrpF* | GGGAACTGCACGCC-AGGGTT-AACCACTCACTA- | [34] |
| **16** | Pba4 | − | *P. atrosepticum* | − | SCRI1043 | *hrpN* | GGGAACCACGCAG-GCAAGA-AAATCACTTAATG- | [34] |
| **17** | Pba5 | − | *P. atrosepticum* | − | SCRI1043 | *hrpW* | GGGAACCACATCA--CACCGCTCTTCACTTAATA- | [34] |
| **18** | Pba6 | − | *P. atrosepticum* | − | SCRI1043 | *dspE* | GGGAACCAGAGGG-GGGAAA-TGACCACTTAACT- | [34] |
| ***Pseudomonas syringae*** | | | | | | | | |
| **19** | Psyr1 | J03681 | *P. syringae* | tomato | PT23 | *avrD* | TGGAACCAAATCC-GTCCCAAAGGCCACACAGAG- | [37] |
| **20** | Psyr2 | J03682 | *P. syringae* | glycinia | − | *avrD* | TGGAACCAAATCC-GTCCCAAAGGCCACACATTT- | [37] |
| **21** | Psyr3 | L11336 | *P. syringae* | phaseolicola | 3121 | *avrD* | TGGAACCAAATCC-GTCCCAAAGGCCACACAGAG- | [37] |
| **22** | Psyr4 | M15194 | *P. syringae* | glycinia | Race 6 | *avrA* | TGAAACCGAAACGGCGTTGC-TTGCCACACAGCA- | [37] |
| **23** | Psyr5 | M21965 | *P. syringae* | glycinia | Race 4 | *avrB* | TGGAACCTAATTC-AGGGTAAATGCCACACAGCT- | [37] |
| **24** | Psyr6 | L20425 | *P. syringae* | tomato | DC3000 | *avrPto* | TGGAACCGATCCGCTCCCTA-TGACCACTCAAGT- | [37] |
| **25** | Psyr7 | Z21715 | *P. syringae* | tomato | JL1065 | *avrRpt2* | GGGAACCCATTCA-TTGTTTGGAACCACCAACGG- | [37] |
| **26** | Psyr8 | X84843 | *P. syringae* | pisi | 870A | *avrPpiB.R3* | GGGAACCACATCA-TGGGTAAAAGCCACGAAGAG- | [37] |
| **27** | Psyr9 | L14926 | *P. syringae* | syringae | 61 | *hopPsyA (hrmA)* | TGGAACCTTATCG-GGAAAATTTGCCACCCACCC- | [37] |
| **28** | Psyr10 | U16119 | *P. syringae* | tomato | PT23 | *avrE* | GGGAACCGGTCGCTGCGCT--TTGCCACTCACTT- | [37] |
| **29** | Psyr11 | AF232006 | *P. syringae* | tomato | DC3000 | *avrE* | TGGAACCC-GCTGGCATTGC-ATGCCACTCATCC- | [37] |
| **30** | Psyr12 | M22219 | *P. syringae* | glycinia | Race 4 | *avrC* | TGGAACCGTTCTG-CA-ACTCGTGCCACTAAGCT- | [37] |
| **31** | Psyr13 | AJ251482 | *P. syringae* | pisi | Race 2 | *avrPpiA1* | GGGAACTCATTT--TCTTTTAAAACCACACATGT- | [37] |
| **32** | Psyr14 | M 2X 67808 | *P. syringae* | maculicola | M2 | *avrRPM1* | GGGAACTCATTT--TCTTTTAAAACCACACATGT- | [37] |
| **33** | Psyr15 | AJ222647 | *P. syringae* | pisi | − | *avrPpiA2.R2* | GGGAACTCATTT--TCTTTTAAAACCACACATGT- | [37] |
| **34** | Psyr16 | M86401 | *P. syringae* | phaseolicola | − | *avrPphB* | TGGAACCGAATGGGTCAGCTG--GACACTTAGAT- | [37] |
| **35** | Psyr17 | AJ277496 | *P. syringae* | pisi | Race 1 (299A) | *avrPpiC2* | GGGAACTGAACC--GCTTATGAAACCACTCATTT- | [37] |
| **36** | Psyr18 | AJ277495 | *P. syringae* | pisi | Race 4A (895A) | *avrPpiG1* | TGGAACCACGGTAGCTTAGC-TGACCACTCAAGG- | [37] |
| **37** | Psyr19 | AJ277494 | *P. syringae* | phaseolicola | Race 4 (1302A) | *avrPphD* | TGGAACCCAAGA--GCCCTTGCGACCACACATTG> | [37] |
| **38** | Psyr20 | AF231453 | *P. syringae* | phaseolicola | 1449A | *avrPphF* | TGGAACCA-GCTACATAGGTATGACCACTGATCT- | [37] |
| **39** | Psyr21 | PSJ224433 | *P. syringae* | phaseolicola | 1302A | *hrpY* | TGGAACCAACTC--GCACGCAAAACCACACAGTT- | [37] |
| **40** | Psyr22 | − | *P. syringae* | syringae | 61 | *hrpK* | TGGAACCAACTC--GCACGCAAAACCACACAGTT- | [37] |
| **41** | Psyr23 | − | *P. syringae* | syringae | 61 | *hrpJ (operon)* | GGGAACCGAACCGCGTCAAT-GACCCACTCAGCG- | [37] |
| **42** | Psyr24 | − | *P. syringae* | syringae | 61 | *hrpU (operon)* | TGGAACTGAAA-T-CGATCCTCGACCACTTAGCA- | [37] |
| **43** | Psyr25 | − | *P. syringae* | syringae | 61 | *hrpC (operon)* | TGGAACCGCTCCACCTGTTT-GCTCCACTCAAGG- | [37] |
| **44** | Psyr26 | L41863 | *P. syringae* | syringae | 61 | *hrpZ (operon)* | TGGAACCGATTCG-CGGACACATGCCACCTAGCT- | [37] |
| **45** | Psyr27 | − | *P. syringae* | syringae | B728a | *hrpZ (operon)* | TGGAACCGATTCG-CGGACGCATGCCACCTAGCT- | [37] |
| **46** | Psyr28 | − | *P. syringae* | tomato | DC3000 | *hrpK* | TGGAACCAACTT--GCACCTTCAACCACACAGTT- | [37] |
| **47** | Psyr29 | − | *P. syringae* | tomato | DC3000 | *hrpJ* | GGGAACTGATCCGGGACCGT-GACCCACTCAGCG- | [37] |
| **48** | Psyr30 | − | *P. syringae* | tomato | DC3000 | *hrpU (operon)* | TGGAACTGAAA-T-CGATGCTCGACCACTTATCA- | [37] |
| **49** | Psyr31 | − | *P. syringae* | tomato | DC3000 | *hrpC (operon)* | TGGAACCGCTCGGCGGGTTT-GCTCCACTCAAGG- | [37] |
| **50** | Psyr32 | − | *P. syringae* | tomato | DC3000 | *hrpZ (operon)* | TGGAACCGTATCG-CAGGCTGCTGCCACCTAGTG- | [37] |
| **51** | Psyr33 | AF268940 | *P. syringae* | phaseolicola | 1302A | *hrpZ (operon)* | TGGAACCGATTTA-AGGGTCGTTACCACCTATCT | [37] |
| **52** | Psyr34 | − | *P. syringae* | − | − | *HrpF03, orf00584* | TGGAACCT-CACGCTTAGTGATGACCACGCATAG- | [37]; Tn5 screen |
| **53** | Psyr35 | − | *P. syringae* | − | − | *HrpF07, hrpW* | GGGAACCGGTCGCTGCGCT--TTGCCACTCACTT- | [37]; Tn5 screen |
| **54** | Psyr36 | − | *P. syringae* | − | − | *HrpF09, orf04808* | TGGAACCAGATCT-CGT-TGCTTGCCACCAAGGC- | [37]; Tn5 screen |
| **55** | Psyr37 | − | *P. syringae* | − | − | *HrpF43, iaal* | CGGAACTGCAAC--GTTGTTTCGGTCACTCAGTG- | [37]; Tn5 screen |
| **56** | Psyr38 | − | *P. syringae* | − | − | *HrpR20, orf11016* | TGGAACCGGACGA-GGCTTT-TTACCACTCAATG- | [37]; Tn5 screen |
| **57** | Psyr39 | − | *P. syringae* | − | − | *HrpR23, AvrPto2* | TGGAACTCTTTCC-TGCTCTTTTGCCACACAGCG- | [37]; Tn5 screen |
| **58** | Psyr40 | − | *P. syringae* | − | − | *HrpR25, orf07885* | CGGAACCGAATC--CATATTTCGACCACCCATCC- | [37]; Tn5 screen |
| **59** | Psyr41 | − | *P. syringae* | − | − | *HrpR26, orf07755* | GGGAACCCTGCGCAGGTCAT-TGACCACTCAGTG- | [37]; Tn5 screen |
| **60** | Psyr42 | − | *P. syringae* | − | − | *HrpR31, orf7cel* | GGGAACCGCATCA-CGT-CTTGAACCACAGAGGA- | [37]; Tn5 screen |
| **61** | Psyr43 | − | *P. syringae* | − | − | *HrpR32, hopPtoA* | TGGAACCGTCAAC-CGATCCGGGACCACACAGCC- | [37]; Tn5 screen |
| **62** | Psyr44 | − | *P. syringae* | − | − | *HrpR34, hrmB/A* | TGGAACCGCCTCG-AGCAGAGGCTCCACTCATTG- | [37]; Tn5 screen |
| **63** | Psyr45 | − | *P. syringae* | − | − | *HrpR36, orf1cel* | GGGAACCGTAACGGCGA-GC-GTGCCACGTAGGG- | [37]; Tn5 screen |
| **64** | Psyr46 | − | *P. syringae* | − | − | *HrpR40, orf2eel* | TGGAACCGATTTC-GAT-GAGTCGCCACACATAA- | [37]; Tn5 screen |
| **65** | Psyr47 | − | *P. syringae* | − | − | *HrpR41, AvrPpiC2* | GGGAACTGAACC--GCTTATGAAACCACTCATTT- | [37]; Tn5 screen |
| **66** | Psyr48 | − | *P. syringae* | − | − | *HrpR55* | CGGAACTCTTTCCCTGCGCTTT--CCACTCAGGG- | [37]; Tn5 screen |
| **67** | Psyr49 | − | *P. syringae* | − | − | *HrpR57, orf00527* | CGGAACCGATCCGGTTGCC--TGGCCACTCAATT- | [37]; Tn5 screen |
| **68** | Psyr50 | − | *P. syringae* | − | − | *HrpR72, AvrPphE* | GGGCACCGACCACGCCGGTA-TCGCCACGCAGAT- | [37]; Tn5 screen |
| **69** | Psyr51 | − | *P. syringae* | − | − | *HrpR85* | AGGAACTCATCACCG-CGAAT-CGCCACTCAGCA- | [37]; Tn5 screen |

50. Gaudriault S, Malandrin L, Paulin JP, Barny MA (1997) DspA, an essential pathogenicity factor of *Erwinia amylovora* showing homology with AvrE of *Pseudomonas syringae*, is secreted via the Hrp secretion pathway in a DspB-dependent way. Mol Microbiol 26: 1057-1069.

51. Giorgi S, Scortichini M (2005) Molecular characterization of *Erwinia amylovora* strains from differenthost plants through RFLP analysis and sequencing of *hrpN* and *dspA/E* genes. Plant Pathol 6:789-798.

52. Bogdanove AJ, Wei ZM, Zhao L, Beer SV (1996) *Erwinia amylovora* secretes harpin via a type III pathway and contains a homolog of *yopN* of *Yersinia* spp. J Bacteriol 178: 1720-1730.

53. Kim JF, Beer SV (1998) HrpW of *Erwinia amylovora*, a new harpin that contains a domain homologous to pectate lyases of a distinct class. J Bacteriol 180: 5203-5210.

54. Gaudriault S, Brisset MN, Barny MA (1998) HrpW of *Erwinia amylovora*, a new Hrp-secreted protein. FEBS Lett 428: 224-228.

55. Kim JF, Wei ZM, Beer SV (1997) The *hrpA* and *hrpC* operons of *Erwinia amylovora* encode components of a type III pathway that secretes harpin. J Bacteriol 179: 1690-1697.

56. Wei ZM, Beer SV (1995) *hrpL* activates *Erwinia amylovora hrp* gene transcription and is a member of the ECF subfamily of sigma factors. J Bacteriol 177: 6201-6210.

57. Kim JF, Ham JH, Bauer DW, Collmer A, Beer SV (1998) The *hrpC* and *hrpN* operons of *Erwinia chrysanthemi* EC16 are flanked by *plcA* and homologs of hemolysin/adhesin genes and accompanying activator/transporter genes. Mol Plant-Microbe Interact 11: 563-567.
