# Supplementary material for: Genome-Wide Identification of HrpL-Regulated Genes in the Necrotrophic Phytopathogen Dickeya dadantii 3937
Source: PLoS One. 2010 Oct 19;5(10):e13472. doi: 10.1371/journal.pone.0013472 (PMC2957411; doi:10.1371/journal.pone.0013472)
Supplement: Table S3 — HMM predictions of the locations of hrp boxes having bit score greater than the optimal prediction threshold 8.5. Columns represent: Feature ID of Dickeya dadantii 3937 ASAP accession ID of version v6b; HMMer bit score for the prediction; distance between prediction and the closest downstream gene; name of closest downstream gene; annotated product of closest downstream gene; orientation of closest downstream gene; orientation of HMMer prediction. (0.13 MB DOC) [file pone.0013472.s003.doc]

| **Feature**  **ID** | **HMMER**  **score** | **d2cds** | **Gene** | **Product** | **Gene**  **Strand** | **Predicted**  **HitStrand** |
| --- | --- | --- | --- | --- | --- | --- |
| **19004** | **23.18** | **41** | ***hrpK*** | **hrpK** | - | - |
| **20784** | **20.94** | **94** | ***hrpN*** | **type III secreted protein HrpN** | - | - |
| **16408** | **20.60** | **48** |  | **acetyltransferase** | + | + |
| **20866** | **20.40** | **59** | ***hrpF*** | **HrpF** | - | - |
| **15579** | **17.43** | **42** | ***hrpJ*** | **type III secretion protein HrpJ** | + | + |
| **19009** | **13.05** | **1** | ***hrpW*** | **type III secreted protein HrpW** | + | + |
| **19593** | **11.01** | **81** | ***hrpA*** | **HrpA** | - | - |
| **20645** | **10.60** | **117** | ***tdk*** | **thymidine kinase** | + | + |
| **20336** | **9.75** | **39** | [***arbF***](http://asap.ahabs.wisc.edu/asap/feature_info.php?FeatureID=20336&LocationID=WIS&SequenceVersionID=41&FeatureDate=20100310235959) | **PTS system beta-glucoside-specific IIA** | - | - |
| **14811** | **8.79** | **128** | ***ytfK*** | **unknown protein** | - | - |
| **14756** | **8.68** | **214** | ***ydgD*** | **putative protease** | + | + |
| 17761 | 8.33 | 230 | *ycfC* | unknown protein | + | + |
| 16971 | 7.96 | 121 | *kdpD* | sensory histidine kinase in two-component regulatory system with KdpE | - | - |
| 16143 | 7.96 | 103 | *glyQ* | glycine tRNA synthetase | - | - |
| 15698 | 7.76 | 1 |  | unknown protein | - | - |
| 16844 | 6.72 | 29 |  | unknown protein | - | - |
| 18791 | 5.63 | 316 |  | periplasmic binding protein | + | + |
| 20104 | 5.08 | 725 | *yddG* | unknown protein | + | + |
| 20517 | 5.01 | 207 | *cycA* | transport of D-alanine | + | + |
| 15773 | 4.90 | 895 |  | unknown protein | - | - |
| 20090 | 4.21 | 174 | *sufA* | SufA protein | - | - |
| 18325 | 4.02 | 212 | *leuS* | leucyl-tRNA synthetase | - | - |
| 17312 | 3.96 | 136 | *ybbO* | short chain dehydrogenase/oxidoreductase | - | - |
| 15043 | 3.67 | 106 |  | unknown protein | + | + |
| 18338 | 3.29 | 142 | *lipB* | lipoate-protein ligase B | - | - |
| 17125 | 3.28 | 57 | *inh* | protease inhibitor | - | - |
| 15649 | 3.20 | 50 | *yhdA* | unknown protein | + | + |
| 19208 | 3.10 | 126 |  | unknown protein | - | - |
| 17538 | 3.08 | 7 | *yaiD* | DNA recombination-dependent growth factor C | - | - |
| 15081 | 2.95 | 210 |  | unknown protein | + | + |
| 16257 | 2.85 | 407 |  | unknown protein | - | - |
| 20198 | 2.79 | 135 | *sfcA* | NAD-linked malate dehydrogenase | - | - |
| 19390 | 2.78 | 229 | *ycbJ* | unknown protein | + | + |
| 19333 | 2.50 | 852 |  | unknown protein | + | + |
| 18016 | 2.36 | 156 |  | sugar ABC transport system, permease | - | - |
| 18173 | 2.36 | 77 | *suhB* | inositol--monophosphatase | + | + |
| 15591 | 2.22 | 217 |  | unknown protein | + | + |
| 15989 | 2.15 | 99 |  | transporter | + | + |
| 18038 | 2.10 | 95 |  | IS1617 transposase | + | + |
| 20837 | 2.05 | 54 | *pelC* | pectate lyase C | + | + |
| 17825 | 2.00 | 246 | *lysC* | aspartokinase III | + | + |
| 46937 | 1.95 | 91 |  | unknown protein | + | + |
| 16000 | 1.78 | 82 |  | amino acid ABC transporter | - | - |
| 18245 | 1.52 | 252 | *yeaK* | unknown protein | - | - |
| 46702 | 1.50 | 484 |  | unknown protein | + | + |
| 16928 | 1.50 | 21 | *rrmB* | tRNA and rRNA cytosine-C5-methylase | + | + |
| 16981 | 1.46 | 125 | *fldA* | flavodoxin 1 | - | - |
| 46571 | 1.39 | 743 |  | TetR family regulator | - | - |
| 17286 | 1.32 | 132 |  | unknown protein | - | - |
| 17452 | 1.28 | 73 |  | hypothetical protein | + | + |
| 19863 | 1.27 | 123 | *cysU* | sulfate ABC transporter | - | - |
| 16478 | 1.26 | 46 |  | putative TetR family regulator | - | - |
| 18210 | 1.25 | 225 |  | unknown protein | + | + |
| 46969 | 1.17 | 8 | [*nagC*](http://asap.ahabs.wisc.edu/asap/feature_info.php?FeatureID=46969&LocationID=WIS&SequenceVersionID=41&FeatureDate=20100310235959) | transcriptional dual regulator | - | - |
| 15031 | 1.14 | 176 | *glpT* | sn-glycerol-3-phosphate permease | + | + |
| 19702 | 1.06 | 315 |  | unknown conserved protein | - | - |
| 17142 | 1.06 | 175 |  | unknown protein | - | - |
| 19050 | 1.06 | 23 |  | methyl-accepting chemotaxis protein | - | - |
| 18830 | 1.03 | 181 |  | unknown protein | + | + |
| 17963 | 0.97 | 436 | *fliD* | filament capping protein | + | + |
| 15100 | 0.87 | 165 | *mrcA* | peptidoglycan synthetase | + | + |
| 46996 | 0.81 | 625 |  | MsbA family ABC transporter | - | - |
| 20049 | 0.81 | 31 | *mfd* | transcription-repair coupling factor | + | + |
| 16619 | 0.79 | 428 |  | unknown protein | - | - |
| 15028 | 0.57 | 110 | *yigL* | hydrolase | - | - |
| 20001 | 0.56 | 200 |  | unknown protein | - | - |
| 16549 | 0.41 | 565 |  | sugar kinase | - | - |
| 16958 | 0.40 | 99 | *gltA* | citrate synthase | - | - |
| 18592 | 0.36 | 137 | *yjjV* | Mg-dependent DNase | + | + |
| 19699 | 0.25 | 849 | *speC* | ornithine decarboxylase isozyme | + | + |
| 19910 | 0.25 | 41 |  | probable zinc-binding oxidoreductase | + | + |
| 14766 | 0.24 | 10 | *lolA* | outer membrane lipoprotein | + | + |
| 14894 | 0.21 | 12 | *rplK* | 50S ribosomal subunit protein L11 | + | + |
