# Supplementary material for: Genome-Wide Identification of HrpL-Regulated Genes in the Necrotrophic Phytopathogen Dickeya dadantii 3937
Source: PLoS One. 2010 Oct 19;5(10):e13472. doi: 10.1371/journal.pone.0013472 (PMC2957411; doi:10.1371/journal.pone.0013472)
Supplement: Table S4 — Genes associated with type III secretion pathway in 3937. (0.12 MB DOC) [file pone.0013472.s004.doc]

| **FeatureID** | **Name** | **Strand** | **Left End** | **Right End** | **Product** |
| --- | --- | --- | --- | --- | --- |
| [15577](http://asap.ahabs.wisc.edu/asap/feature_info.php?FeatureID=ABF-0015577&LocationID=WIS&SequenceVersionID=41&FeatureDate=20100426235959) | [*hrpL*](http://asap.ahabs.wisc.edu/asap/feature_info.php?FeatureID=15577&LocationID=WIS&SequenceVersionID=41&FeatureDate=20100426235959) | - | 2650274 | 2650828 | RNA polymerase sigma-54 factor RpoN |
| [**15579**](http://asap.ahabs.wisc.edu/asap/feature_info.php?FeatureID=ABF-0015579&LocationID=WIS&SequenceVersionID=41&FeatureDate=20100426235959) | [***hrpJ***](http://asap.ahabs.wisc.edu/asap/feature_info.php?FeatureID=15579&LocationID=WIS&SequenceVersionID=41&FeatureDate=20100426235959) | **+** | **2651151** | **2652320** | **T3S protein** |
| [15580](http://asap.ahabs.wisc.edu/asap/feature_info.php?FeatureID=ABF-0015580&LocationID=WIS&SequenceVersionID=41&FeatureDate=20100426235959) | [*hrcV*](http://asap.ahabs.wisc.edu/asap/feature_info.php?FeatureID=15580&LocationID=WIS&SequenceVersionID=41&FeatureDate=20100426235959) | + | 2652324 | 2654426 | T3S inner membrane channel protein |
| [15581](http://asap.ahabs.wisc.edu/asap/feature_info.php?FeatureID=ABF-0015581&LocationID=WIS&SequenceVersionID=41&FeatureDate=20100426235959) | [*hrpQ*](http://asap.ahabs.wisc.edu/asap/feature_info.php?FeatureID=15581&LocationID=WIS&SequenceVersionID=41&FeatureDate=20100426235959) | + | 2654438 | 2655409 | HrpQ |
| [15582](http://asap.ahabs.wisc.edu/asap/feature_info.php?FeatureID=ABF-0015582&LocationID=WIS&SequenceVersionID=41&FeatureDate=20100426235959) | [*hrcN*](http://asap.ahabs.wisc.edu/asap/feature_info.php?FeatureID=15582&LocationID=WIS&SequenceVersionID=41&FeatureDate=20100426235959) | + | 2655406 | 2656788 | ATPase component HrcN |
| [15583](http://asap.ahabs.wisc.edu/asap/feature_info.php?FeatureID=ABF-0015583&LocationID=WIS&SequenceVersionID=41&FeatureDate=20100426235959) | [*hrpO*](http://asap.ahabs.wisc.edu/asap/feature_info.php?FeatureID=15583&LocationID=WIS&SequenceVersionID=41&FeatureDate=20100426235959) | + | 2656775 | 2657254 | HrpO |
| [15584](http://asap.ahabs.wisc.edu/asap/feature_info.php?FeatureID=ABF-0015584&LocationID=WIS&SequenceVersionID=41&FeatureDate=20100426235959) | [*hrpP*](http://asap.ahabs.wisc.edu/asap/feature_info.php?FeatureID=15584&LocationID=WIS&SequenceVersionID=41&FeatureDate=20100426235959) | + | 2657251 | 2657775 | HrpP |
| [15585](http://asap.ahabs.wisc.edu/asap/feature_info.php?FeatureID=ABF-0015585&LocationID=WIS&SequenceVersionID=41&FeatureDate=20100426235959) | [*hrcQ*](http://asap.ahabs.wisc.edu/asap/feature_info.php?FeatureID=15585&LocationID=WIS&SequenceVersionID=41&FeatureDate=20100426235959) | + | 2657772 | 2658920 | HrcQ |
| [15586](http://asap.ahabs.wisc.edu/asap/feature_info.php?FeatureID=ABF-0015586&LocationID=WIS&SequenceVersionID=41&FeatureDate=20100426235959) | [*hrcR*](http://asap.ahabs.wisc.edu/asap/feature_info.php?FeatureID=15586&LocationID=WIS&SequenceVersionID=41&FeatureDate=20100426235959) | + | 2658917 | 2659570 | T3S inner membrane protein |
| [15587](http://asap.ahabs.wisc.edu/asap/feature_info.php?FeatureID=ABF-0015587&LocationID=WIS&SequenceVersionID=41&FeatureDate=20100426235959) | [*hrcS*](http://asap.ahabs.wisc.edu/asap/feature_info.php?FeatureID=15587&LocationID=WIS&SequenceVersionID=41&FeatureDate=20100426235959) | + | 2659578 | 2659838 | T3S inner membrane protein |
| [15588](http://asap.ahabs.wisc.edu/asap/feature_info.php?FeatureID=ABF-0015588&LocationID=WIS&SequenceVersionID=41&FeatureDate=20100426235959) | [*hrcT*](http://asap.ahabs.wisc.edu/asap/feature_info.php?FeatureID=15588&LocationID=WIS&SequenceVersionID=41&FeatureDate=20100426235959) | + | 2659846 | 2660649 | T3S inner membrane protein |
| [15589](http://asap.ahabs.wisc.edu/asap/feature_info.php?FeatureID=ABF-0015589&LocationID=WIS&SequenceVersionID=41&FeatureDate=20100426235959) | [*hrcU*](http://asap.ahabs.wisc.edu/asap/feature_info.php?FeatureID=15589&LocationID=WIS&SequenceVersionID=41&FeatureDate=20100426235959) | + | 2660689 | 2661768 | T3S inner membrane protein |
| [17117](http://asap.ahabs.wisc.edu/asap/feature_info.php?FeatureID=ABF-0017117&LocationID=WIS&SequenceVersionID=41&FeatureDate=20100426235959) | [*srfC*](http://asap.ahabs.wisc.edu/asap/feature_info.php?FeatureID=17117&LocationID=WIS&SequenceVersionID=41&FeatureDate=20100426235959) | + | 2446182 | 2448686 | Virulence factor SrfC homolog |
| [17118](http://asap.ahabs.wisc.edu/asap/feature_info.php?FeatureID=ABF-0017118&LocationID=WIS&SequenceVersionID=41&FeatureDate=20100426235959) | [*srfB*](http://asap.ahabs.wisc.edu/asap/feature_info.php?FeatureID=17118&LocationID=WIS&SequenceVersionID=41&FeatureDate=20100426235959) | + | 2443195 | 2446185 | SrfB |
| [17119](http://asap.ahabs.wisc.edu/asap/feature_info.php?FeatureID=ABF-0017119&LocationID=WIS&SequenceVersionID=41&FeatureDate=20100426235959) | [*srfA*](http://asap.ahabs.wisc.edu/asap/feature_info.php?FeatureID=17119&LocationID=WIS&SequenceVersionID=41&FeatureDate=20100426235959) | + | 2441876 | 2443192 | putative virulence factor |
| [**19004**](http://asap.ahabs.wisc.edu/asap/feature_info.php?FeatureID=ABF-0019004&LocationID=WIS&SequenceVersionID=41&FeatureDate=20100426235959) | [***hrpK***](http://asap.ahabs.wisc.edu/asap/feature_info.php?FeatureID=19004&LocationID=WIS&SequenceVersionID=41&FeatureDate=20100426235959) | **-** | **2517540** | **2519255** | **Pathogenicity locus protein hrpK** |
| [**19009**](http://asap.ahabs.wisc.edu/asap/feature_info.php?FeatureID=ABF-0019009&LocationID=WIS&SequenceVersionID=41&FeatureDate=20100426235959) | [***hrpW***](http://asap.ahabs.wisc.edu/asap/feature_info.php?FeatureID=19009&LocationID=WIS&SequenceVersionID=41&FeatureDate=20100426235959) | **+** | **2514597** | **2516306** | **type III secreted protein** |
| [19012](http://asap.ahabs.wisc.edu/asap/feature_info.php?FeatureID=ABF-0019012&LocationID=WIS&SequenceVersionID=41&FeatureDate=20100426235959) | [*dspE*](http://asap.ahabs.wisc.edu/asap/feature_info.php?FeatureID=19012&LocationID=WIS&SequenceVersionID=41&FeatureDate=20100426235959) | - | 2508322 | 2513199 | DspE |
| [19013](http://asap.ahabs.wisc.edu/asap/feature_info.php?FeatureID=ABF-0019013&LocationID=WIS&SequenceVersionID=41&FeatureDate=20100426235959) | [*dspF*](http://asap.ahabs.wisc.edu/asap/feature_info.php?FeatureID=19013&LocationID=WIS&SequenceVersionID=41&FeatureDate=20100426235959) | - | 2507818 | 2508285 | DspF |
| [19585](http://asap.ahabs.wisc.edu/asap/feature_info.php?FeatureID=ABF-0019585&LocationID=WIS&SequenceVersionID=41&FeatureDate=20100426235959) | [*ORF3*](http://asap.ahabs.wisc.edu/asap/feature_info.php?FeatureID=19585&LocationID=WIS&SequenceVersionID=41&FeatureDate=20100426235959) | + | 2640889 | 2641887 | hypothetical protein |
| [19586](http://asap.ahabs.wisc.edu/asap/feature_info.php?FeatureID=ABF-0019586&LocationID=WIS&SequenceVersionID=41&FeatureDate=20100426235959) | [*ORF4*](http://asap.ahabs.wisc.edu/asap/feature_info.php?FeatureID=19586&LocationID=WIS&SequenceVersionID=41&FeatureDate=20100426235959) | - | 2641898 | 2643211 | Membrane-bound lytic murein transglycosylase B |
| [19587](http://asap.ahabs.wisc.edu/asap/feature_info.php?FeatureID=ABF-0019587&LocationID=WIS&SequenceVersionID=41&FeatureDate=20100426235959) | [*hrpE*](http://asap.ahabs.wisc.edu/asap/feature_info.php?FeatureID=19587&LocationID=WIS&SequenceVersionID=41&FeatureDate=20100426235959) | - | 2643472 | 2644074 | HrpE |
| [19588](http://asap.ahabs.wisc.edu/asap/feature_info.php?FeatureID=ABF-0019588&LocationID=WIS&SequenceVersionID=41&FeatureDate=20100426235959) | [*hrpD*](http://asap.ahabs.wisc.edu/asap/feature_info.php?FeatureID=19588&LocationID=WIS&SequenceVersionID=41&FeatureDate=20100426235959) | - | 2644167 | 2644817 | HrpD |
| [19590](http://asap.ahabs.wisc.edu/asap/feature_info.php?FeatureID=ABF-0019590&LocationID=WIS&SequenceVersionID=41&FeatureDate=20100426235959) | [*hrcJ*](http://asap.ahabs.wisc.edu/asap/feature_info.php?FeatureID=19590&LocationID=WIS&SequenceVersionID=41&FeatureDate=20100426235959) | - | 2644763 | 2645473 | lipoprotein |
| [19592](http://asap.ahabs.wisc.edu/asap/feature_info.php?FeatureID=ABF-0019592&LocationID=WIS&SequenceVersionID=41&FeatureDate=20100426235959) | [*hrpB*](http://asap.ahabs.wisc.edu/asap/feature_info.php?FeatureID=19592&LocationID=WIS&SequenceVersionID=41&FeatureDate=20100426235959) | - | 2645632 | 2646033 | HrpB |
| [**19593**](http://asap.ahabs.wisc.edu/asap/feature_info.php?FeatureID=ABF-0019593&LocationID=WIS&SequenceVersionID=41&FeatureDate=20100426235959) | [***hrpA***](http://asap.ahabs.wisc.edu/asap/feature_info.php?FeatureID=19593&LocationID=WIS&SequenceVersionID=41&FeatureDate=20100426235959) | **-** | **2646081** | **2646284** | **Hrp pili protein hrpA** |
| [19594](http://asap.ahabs.wisc.edu/asap/feature_info.php?FeatureID=ABF-0019594&LocationID=WIS&SequenceVersionID=41&FeatureDate=20100426235959) | [*hrpS*](http://asap.ahabs.wisc.edu/asap/feature_info.php?FeatureID=19594&LocationID=WIS&SequenceVersionID=41&FeatureDate=20100426235959) | - | 2646451 | 2647434 | HrpS type III protein secretion system regulator |
| [19598](http://asap.ahabs.wisc.edu/asap/feature_info.php?FeatureID=ABF-0019598&LocationID=WIS&SequenceVersionID=41&FeatureDate=20100426235959) | [*hrpY*](http://asap.ahabs.wisc.edu/asap/feature_info.php?FeatureID=19598&LocationID=WIS&SequenceVersionID=41&FeatureDate=20100426235959) | - | 2647876 | 2648517 | LuxR family DNA-binding response regulator |
| [19600](http://asap.ahabs.wisc.edu/asap/feature_info.php?FeatureID=ABF-0019600&LocationID=WIS&SequenceVersionID=41&FeatureDate=20100426235959) | [*hrpX*](http://asap.ahabs.wisc.edu/asap/feature_info.php?FeatureID=19600&LocationID=WIS&SequenceVersionID=41&FeatureDate=20100426235959) | - | 2648548 | 2650020 | type III protein secretion system sensor kinase |
| [20783](http://asap.ahabs.wisc.edu/asap/feature_info.php?FeatureID=ABF-0020783&LocationID=WIS&SequenceVersionID=41&FeatureDate=20100426235959) | [*hrpV*](http://asap.ahabs.wisc.edu/asap/feature_info.php?FeatureID=20783&LocationID=WIS&SequenceVersionID=41&FeatureDate=20100426235959) | - | 2635568 | 2635807 | HrpV |
| [**20784**](http://asap.ahabs.wisc.edu/asap/feature_info.php?FeatureID=ABF-0020784&LocationID=WIS&SequenceVersionID=41&FeatureDate=20100426235959) | [***hrpN***](http://asap.ahabs.wisc.edu/asap/feature_info.php?FeatureID=20784&LocationID=WIS&SequenceVersionID=41&FeatureDate=20100426235959) | **-** | **2634379** | **2635407** | **Harpin hrpN** |
| [20863](http://asap.ahabs.wisc.edu/asap/feature_info.php?FeatureID=ABF-0020863&LocationID=WIS&SequenceVersionID=41&FeatureDate=20100426235959) | [*hrpT*](http://asap.ahabs.wisc.edu/asap/feature_info.php?FeatureID=20863&LocationID=WIS&SequenceVersionID=41&FeatureDate=20100426235959) | - | 2635975 | 2636163 | HrpT precursor |
| [20864](http://asap.ahabs.wisc.edu/asap/feature_info.php?FeatureID=ABF-0020864&LocationID=WIS&SequenceVersionID=41&FeatureDate=20100426235959) | [*hrcC*](http://asap.ahabs.wisc.edu/asap/feature_info.php?FeatureID=20864&LocationID=WIS&SequenceVersionID=41&FeatureDate=20100426235959) | - | 2636198 | 2638267 | T3S outermembrane pore forming protein |
| [20865](http://asap.ahabs.wisc.edu/asap/feature_info.php?FeatureID=ABF-0020865&LocationID=WIS&SequenceVersionID=41&FeatureDate=20100426235959) | [*hrpG*](http://asap.ahabs.wisc.edu/asap/feature_info.php?FeatureID=20865&LocationID=WIS&SequenceVersionID=41&FeatureDate=20100426235959) | - | 2638260 | 2638694 | HrpG precursor |
| [**20866**](http://asap.ahabs.wisc.edu/asap/feature_info.php?FeatureID=ABF-0020866&LocationID=WIS&SequenceVersionID=41&FeatureDate=20100426235959) | [***hrpF***](http://asap.ahabs.wisc.edu/asap/feature_info.php?FeatureID=20866&LocationID=WIS&SequenceVersionID=41&FeatureDate=20100426235959) | **-** | **2638681** | **2638908** | **HrpF** |
| [20867](http://asap.ahabs.wisc.edu/asap/feature_info.php?FeatureID=ABF-0020867&LocationID=WIS&SequenceVersionID=41&FeatureDate=20100426235959) | [*plcA*](http://asap.ahabs.wisc.edu/asap/feature_info.php?FeatureID=20867&LocationID=WIS&SequenceVersionID=41&FeatureDate=20100426235959) | - | 2639126 | 2640175 | Extracellular phospholipase C |
| [47134](http://asap.ahabs.wisc.edu/asap/feature_info.php?FeatureID=ABF-0047134&LocationID=WIS&SequenceVersionID=41&FeatureDate=20100426235959) | [*ORF2*](http://asap.ahabs.wisc.edu/asap/feature_info.php?FeatureID=47134&LocationID=WIS&SequenceVersionID=41&FeatureDate=20100426235959) | + | 2640512 | 2640730 | hypothetical protein |

Table was generated based on the annotation in ASAP website. Feature ID: *Dickeya dadantii* 3937 ASAP accession ID of version v6b. T3S: type III secretion. Bold ones are the common T3SS genes predicted by HMM and identified using microarray.
