# Supplementary figures and images for: Genome-Wide Identification of HrpL-Regulated Genes in the Necrotrophic Phytopathogen Dickeya dadantii 3937
Source: PLoS One. 2010 Oct 19;5(10):e13472. doi: 10.1371/journal.pone.0013472 (PMC2957411; doi:10.1371/journal.pone.0013472)

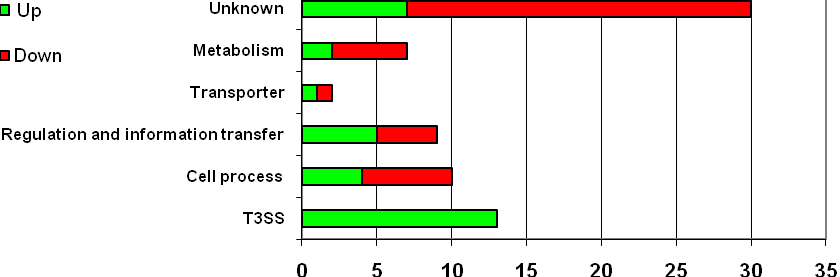

Supplement: Figure S1 — Functional categories of HrpL-regulated genes by comparing the transcriptome profiles of Dickeya dadantii 3937 wild-type and its hrpL mutant in T3SS inducing minimal medium after 6-h post inoculation at 28oC. (0.11 MB DOC) [file pone.0013472.s005.doc]

**
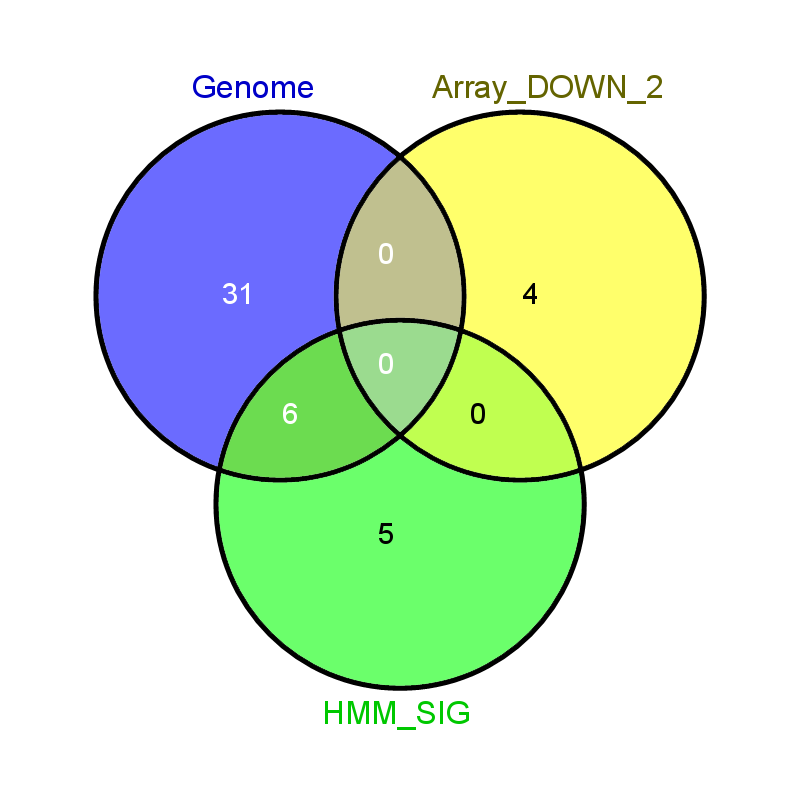

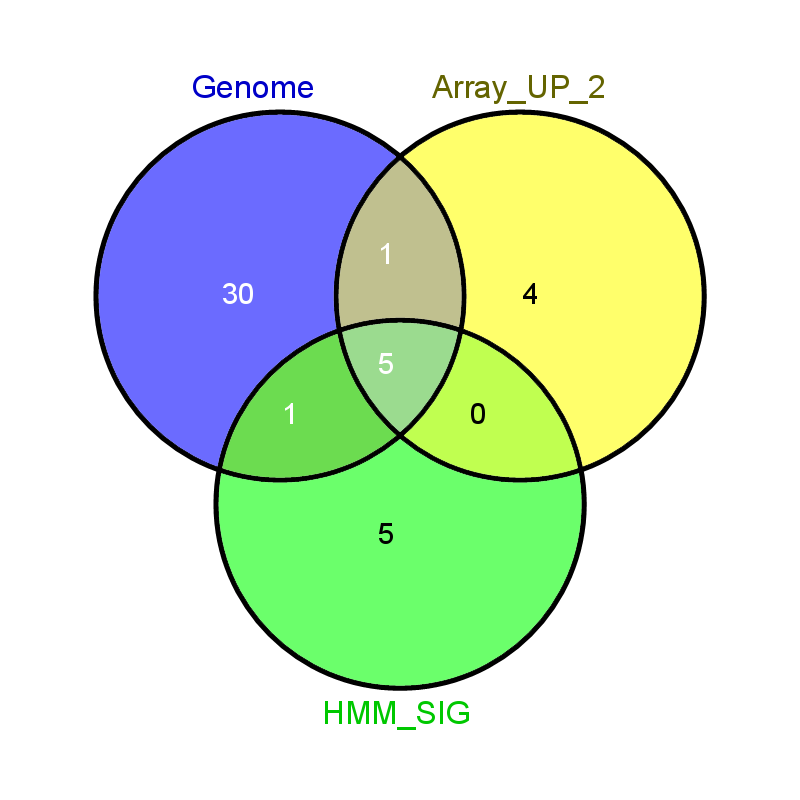

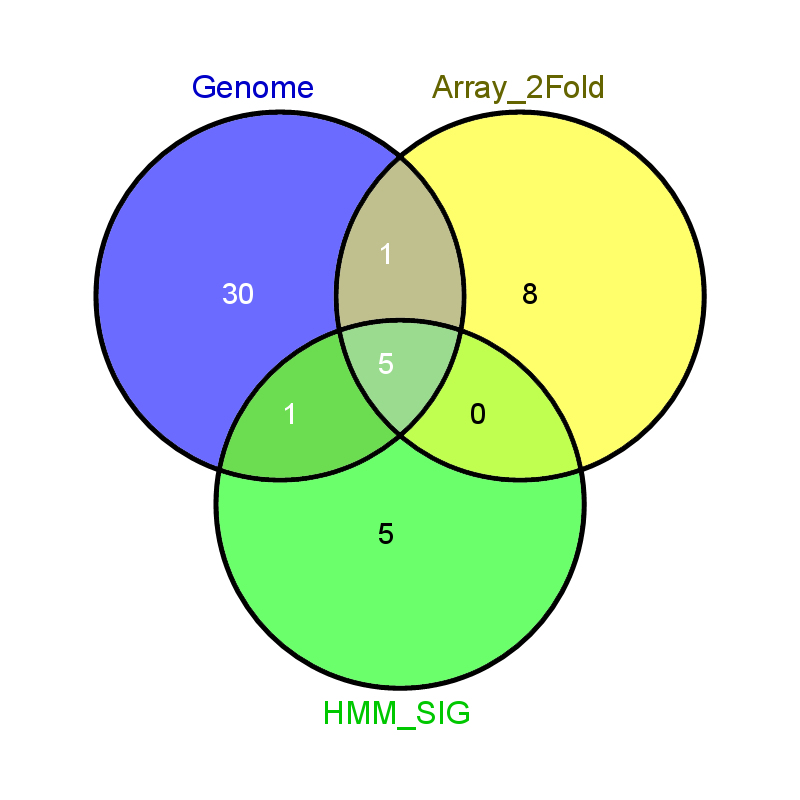
**


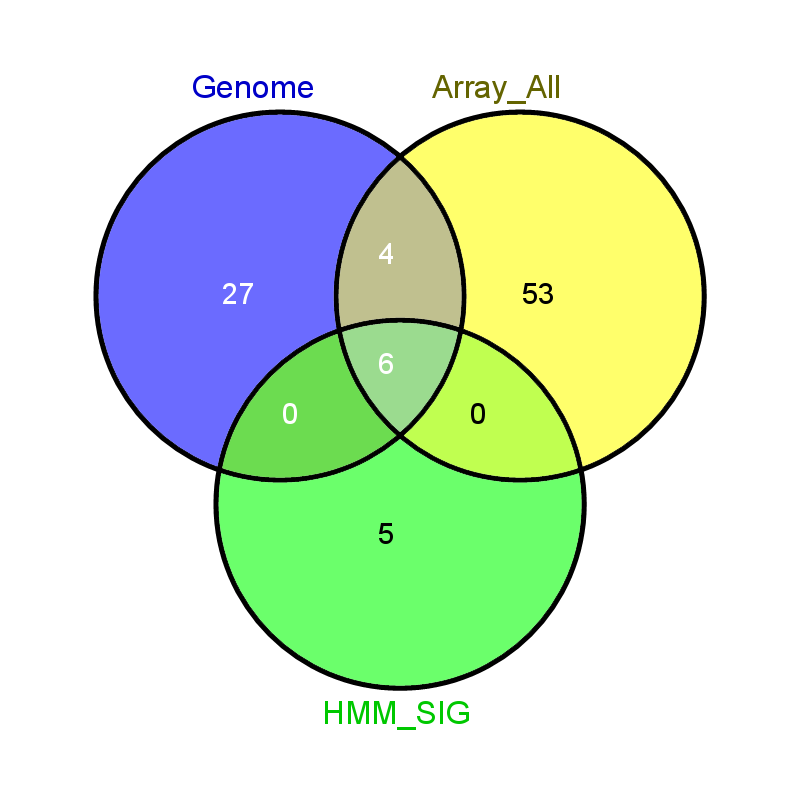

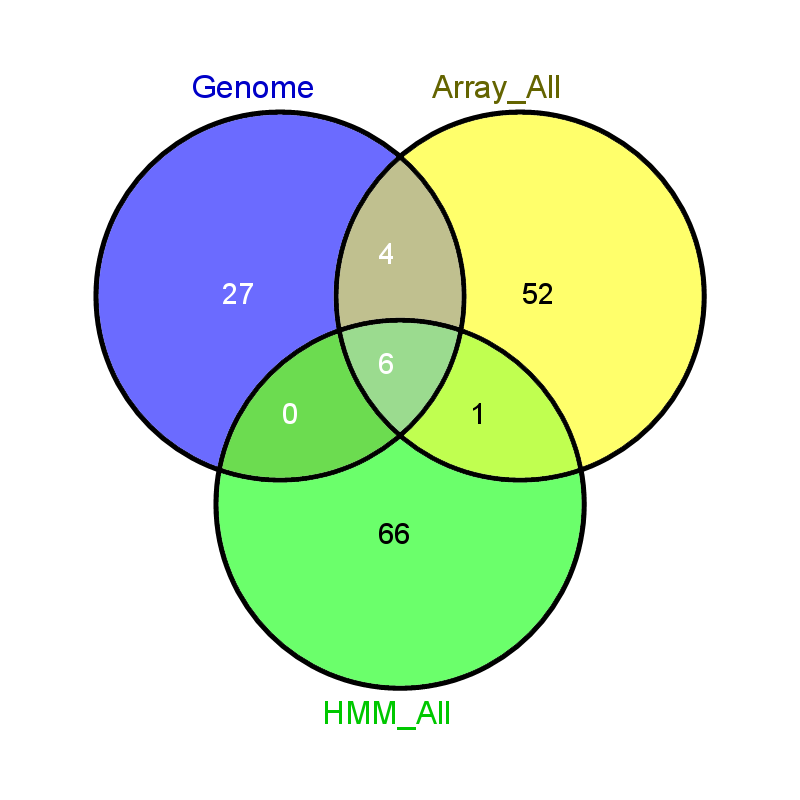

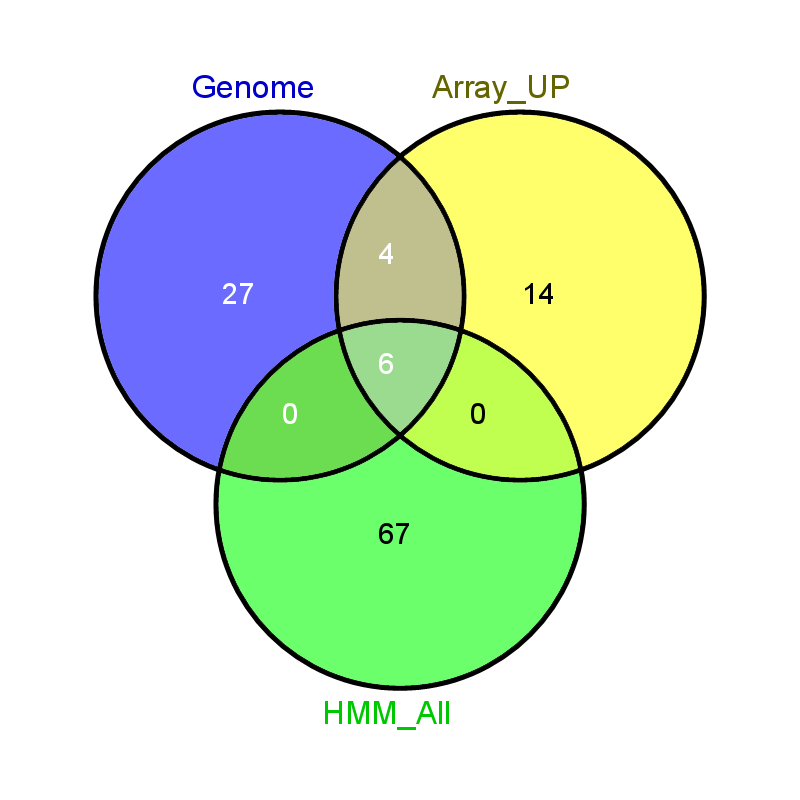

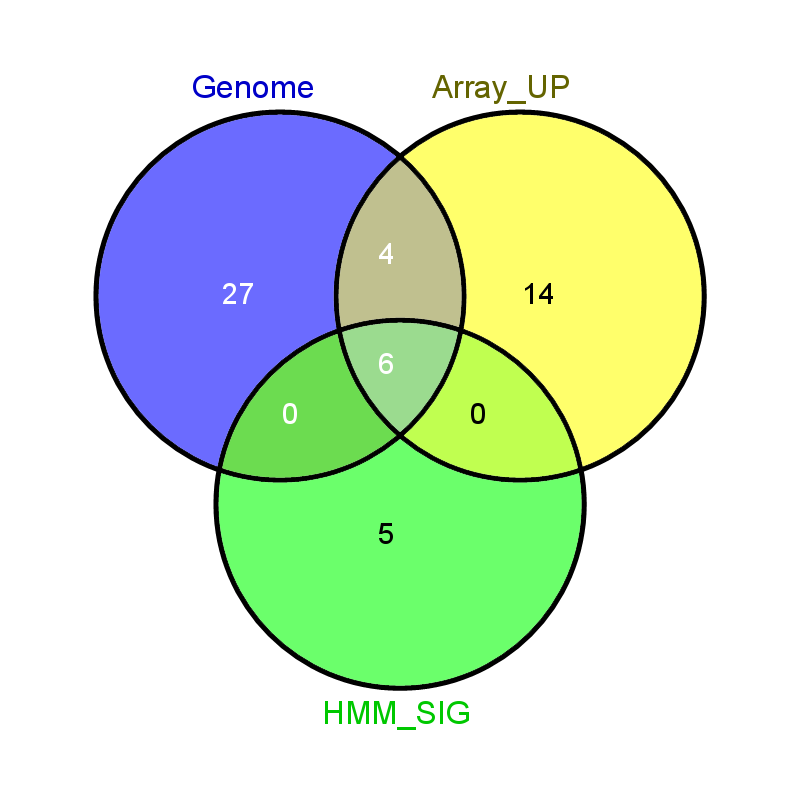


**15579**

**19004**

**19009**

**19593**

**20784**

**20866**

**15584**

**15585**

**19012**

**20865**

**17825**


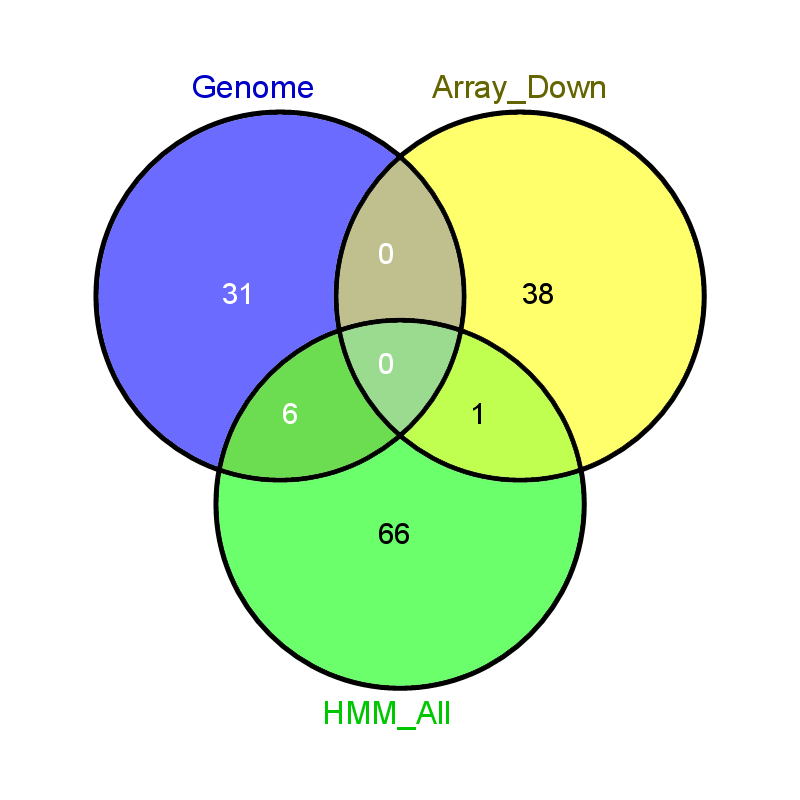

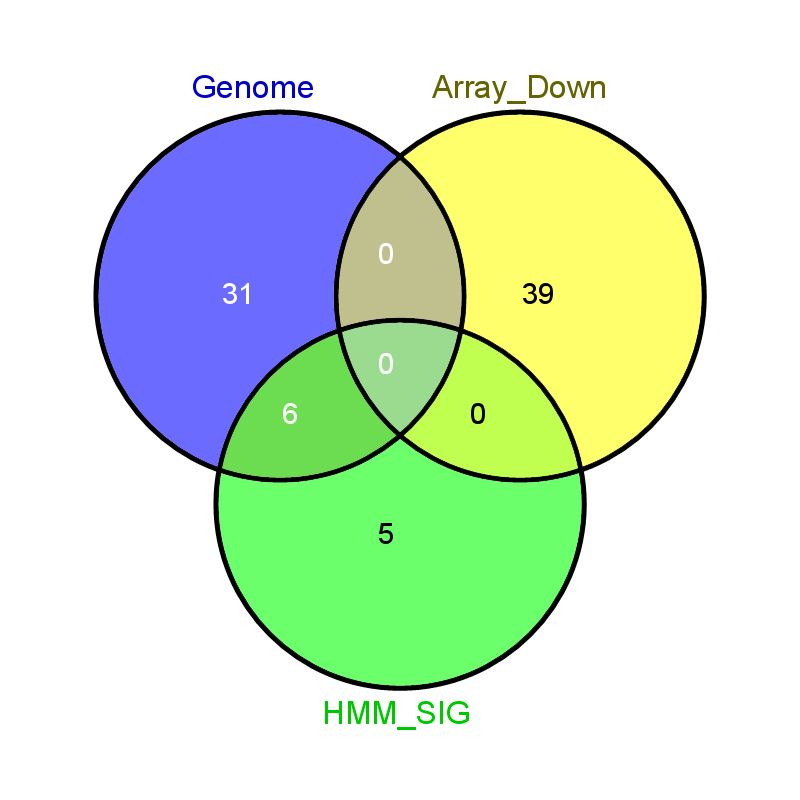

Supplement: Figure S2 — Correlation among the T3SS genes in 3937 genome, HMM prediction, and microarray results. Genome: Dickeya dadantii 3937 T3SS gene ASAP accession ID of version v6b based on the annotation in ASAP website (Supplemental Table 4). Array_All: all the HrpL-regulated genes from microarray experiment with a cut-off value of 1.5-fold (Table 1, 2). Array_UP: all the HrpL-upregulated genes (Table 1). Array_DOWN: all the HrpL-downregulated genes (Table 2). Array_2Fold: all the HrpL-regulated genes with cut-off value of 2-fold changes (Bold ones in Table 1, 2). Array_UP_2: HrpL-upregulated genes with at least 2-fold changes (Bold ones in Table 1). Array_DOWN_2: HrpL-downregulated genes with at least 2-fold changes (Bold ones in Table 2). HMM_All: all the genes predicted by HMM (Supplemental Table 3). HMM_SIG: HMM predicted genes with HMM score greater than 8.5 (Bold ones in Supplemental Table 3). (0.48 MB DOC) [file pone.0013472.s006.doc]
